# Arsenic-induced nephrotoxicity: Mechanisms, biomarkers, and preventive strategies for global health

**DOI:** 10.14202/vetworld.2025.2136-2157

**Published:** 2025-07-30

**Authors:** Preethi Lavina Concessao, Jay Prakash

**Affiliations:** Department of Basic Medical Sciences, Division of Physiology, Manipal Academy of Higher Education, Manipal, Karnataka, India

**Keywords:** arsenic, biomarkers, mechanisms, nephrotoxicity, oxidative stress, therapeutic

## Abstract

Arsenic exposure remains a critical global health concern, with growing evidence linking it to significant kidney dysfunction. This review examines the underlying mechanisms of arsenic-induced nephrotoxicity, including oxidative stress, mitochondrial dysfunction, inflammation, and programmed cell death, which collectively contribute to damage in the glomeruli and renal tubules. Chronic exposure is associated with proteinuria, renal impairment, and an increased risk of chronic kidney disease (CKD). Emerging biomarkers such as β2-microglobulin, kidney injury molecule-1, and neutrophil gelatinase-associated lipocalin have shown promise in detecting arsenic-related renal damage earlier and with greater specificity than traditional markers like serum creatinine. Preventive strategies – such as advanced water purification systems and antioxidant supplementation with agents such as vitamin C, selenium, and curcumin – alongside public health policies targeting arsenic monitoring and regulation, are essential to mitigate exposure risks. Continued research into diagnostic and therapeutic innovations is crucial for reducing the burden of arsenic-induced kidney disease. A deeper understanding of arsenic’s nephrotoxic pathways will support global efforts to protect renal health and strengthen environmental health initiatives.

## INTRODUCTION

Arsenic is a naturally occurring metalloid that is widely distributed in the Earth’s crust. In the environment, arsenic predominantly exists in two oxidation states: Trivalent arsenite (As^3+^) and pentavalent arsenate (As^5+^), with arsenite being more toxic than arsenate. Trivalent arsenic is more prevalent than pentavalent arsenic in groundwater [1]. Arsenic contamination, particularly in groundwater, poses a major threat to environmental and public health in countries such as Bangladesh, India, China, and the United States [2, 3]. Sources of contamination include both natural processes (e.g., volcanic activity and rock weathering) and anthropogenic activities (e.g., mining, well drilling, and fossil fuel combustion) [4–6]. As a result, arsenic contamination presents a significant challenge to both environmental integrity and global public health.

Humans are primarily exposed to arsenic through contaminated drinking water, inhalation of arsenic-laden particulates, and dermal absorption [7]. Arsenic contamination in drinking water can arise from various sources, including natural mineral deposits, arsenical pesticides, and improper chemical disposal. Exposure can also occur through non-water sources, such as food grown with arsenic-contaminated water or cultivated in polluted soil, which poses a substantial health threat, particularly in regions with affected groundwater.

Long-term arsenic exposure – primarily through rice consumption or occupational inhalation in mining and agriculture – can damage the kidneys, disrupt metabolic processes, alter the gut microbiota, and significantly increase the risk of cancer (including lung cancer), cardiovascular disease, diabetes, hypertension, cognitive impairment, reproductive toxicity, and developmental issues in children [8, 9]. In addition, the presence of arsenic in certain medications and herbal remedies – whether intentional or due to contamination – further elevates the risk of cancer and organ damage. Rigorous regulatory oversight is therefore essential to mitigate these health hazards [10, 11].

To reduce health risks, the World Health Organization has established a permissible limit for arsenic in drinking water at 10 μg/L, with an upper limit of 50 μg/L in some regions [12]. Despite these recommendations, it is estimated that 94–220 million people worldwide are chronically exposed to elevated groundwater arsenic levels, and a sizable portion likely suffers from exposure-related health problems (Table 1) [13–15].

**Table 1:** Relative arsenic exposure by route–impact on the kidney [13–15].

Route	Source	Relative exposure level	Risk
Dietary	Rice, water, and food crops	High	Major contributor to kidney damage
Inhalation	Mining, smelting, and pesticides	Moderate	Moderate contributor to kidney damage; occupational hazard
Dermal	Contaminated water and soil	Low	Minimal nephrotoxic effect

Arsenic exposure leads to multisystem toxicity, with significant accumulation in the kidneys, lungs, skin, liver, and bladder [16–19]. Maternal arsenic exposure has been associated with adverse pregnancy outcomes, including increased infant mortality and delayed developmental milestones. Acute toxicity is typically characterized by gastrointestinal symptoms such as vomiting, abdominal pain, diarrhea, and muscle cramps [20].

Considering the widespread distribution of arsenic and its serious health implications, its impact on specific organ systems – particularly the kidneys – must be recognized. As recipients of 20%–25% of the cardiac output, the kidneys are critical for arsenic filtration and excretion, making them especially vulnerable to its toxic effects [21, 22]. Understanding the nephrotoxic mechanisms of arsenic is therefore essential for addressing its role in chronic kidney disease (CKD) and related renal complications.

Although numerous studies have explored the toxicological effects of arsenic exposure, there remains a significant gap in understanding its organ-specific toxicity, particularly its nephrotoxic mechanisms. Most existing research has focused on general systemic toxicity or on carcinogenic outcomes, with limited emphasis on the cellular and molecular pathways that underpin arsenic-induced renal damage. Moreover, conventional renal function markers such as serum creatinine and blood urea nitrogen are often insufficiently sensitive to detect early-stage arsenic-induced kidney injury. While emerging biomarkers such as kidney injury molecule-1 (KIM-1), neutrophil gelatinase-associated lipocalin (NGAL), and β2-microglobulin (β2M) have shown promise, their clinical utility remains under-validated, especially in populations with chronic low-dose exposure. In addition, there is a paucity of integrated research combining molecular mechanisms, biomarker validation, and environmental exposure data to form a comprehensive understanding of arsenic nephrotoxicity. Preventive and therapeutic strategies, including antioxidant and herbal interventions, are often studied in isolation without being linked to individual genetic susceptibility or long-term outcomes. Furthermore, under-researched regions and vulnerable populations continue to be neglected in global arsenic mitigation strategies, leading to insufficient data to support targeted interventions.

This review aims to comprehensively synthesize current knowledge on the mechanisms, biomarkers, and preventive strategies related to arsenic-induced nephrotoxicity. It seeks to elucidate the molecular and cellular pathways through which arsenic damages renal structures, with a focus on oxidative stress, mitochondrial dysfunction, apoptosis, ferroptosis, and inflammation. The review also evaluates novel and emerging biomarkers that offer improved sensitivity and specificity for the early detection of kidney injury due to arsenic exposure. In addition, it highlights therapeutic interventions – including antioxidant supplementation, herbal remedies, and gene-environment interaction approaches – that show potential in mitigating renal damage. By identifying knowledge gaps and emphasizing the importance of personalized medicine, biomarker validation, and underrepresented regions in arsenic research, this review provides a foundation for future studies and informs global public health strategies aimed at preventing arsenic-related kidney disease.

A systematic literature search was conducted using databases such as Scopus, PubMed, MEDLINE, and Google Scholar. Both animal and human studies that were published were evaluated and cited according to their relevance to the discussion. Only peer-reviewed articles published in English were included in the review. Articles were selected based on relevance to the specified keywords, with screening performed on titles, abstracts, and methodologies. 200 articles were shortlisted, and those deemed suitable for the current review were selected.

## ARSENIC METABOLISM IN THE HUMAN BODY

Arsenic primarily exists in drinking water in two inorganic forms: trivalent arsenite [iAs(III)] and pentavalent arsenate [iAs(V)], with iAs(III) being more cytotoxic [23–25]. Once absorbed through the gastrointestinal tract, respiratory system, or skin, arsenic enters systemic circulation predominantly by binding to erythrocytes and subsequently accumulates in organs such as the liver, kidneys, lungs, spleen, and keratin-rich tissues such as the skin and hair.

**Figure 1:**
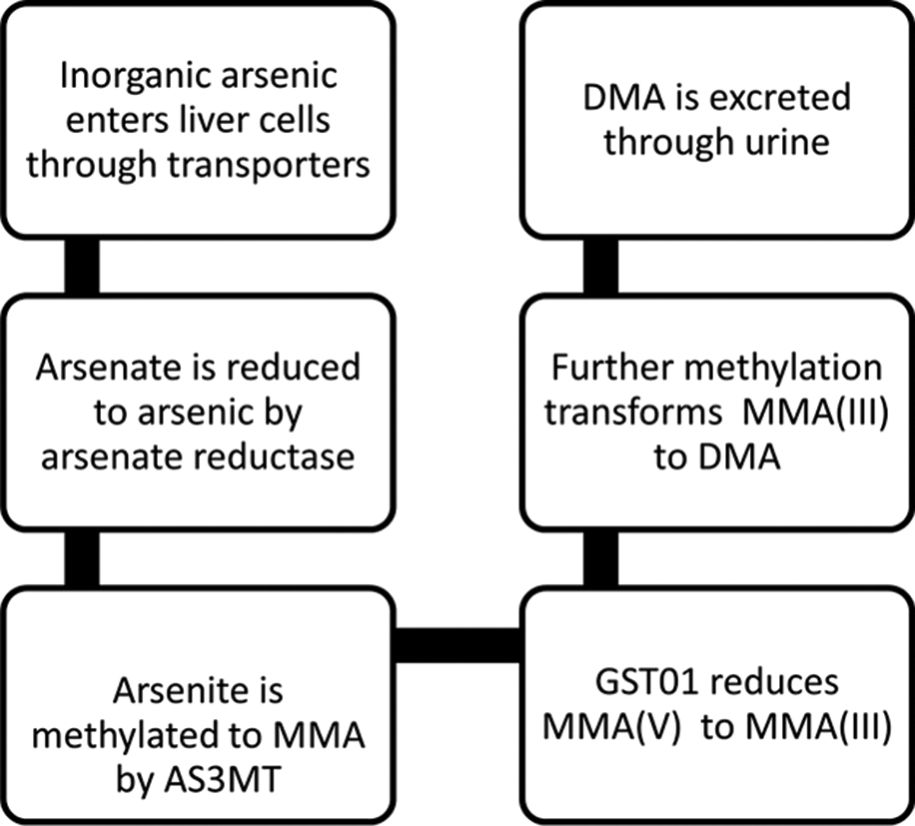
The arsenic metabolism pathway [29].

In the liver, iAs(V) is reduced to iAs(III) by the enzyme arsenate reductase. Cellular uptake of iAs(III) occurs through aquaglyceroporins (AQP7 and AQP9) and glucose transporter 1, while iAs(V) is transported through phosphate channels [26]. Subsequent hepatic methylation, catalyzed by arsenite methyltransferase (AS3MT) and glutathione S-transferase omega 1 (GSTO1), produces the metabolites: monomethylarsonous acid [MMA(III)], monomethylarsonic acid [MMA(V)], dimethylarsinous acid [DMA(III)], and dimethylarsinic acid [DMA(V)] [27, 28]. These metabolites are primarily excreted through urine, with minor elimination through feces, perspiration, and epithelial shedding (Figure 1) [29].

## MECHANISMS OF ARSENIC TOXICITY

Arsenic induces toxicity through a variety of biochemical mechanisms, including oxidative stress, enzyme inhibition, and genotoxicity. It promotes the generation of reactive oxygen species (ROS), resulting in lipid peroxidation, DNA strand breaks, and mitochondrial dysfunction. In addition, arsenic binds to sulfhydryl groups on proteins, notably inhibiting key enzymes such as pyruvate dehydrogenase and impairing adenosine triphosphate (ATP) production [30].

Arsenic also disrupts epigenetic regulation by altering DNA methylation, which can lead to aberrant gene expression and potential carcinogenesis. Furthermore, it activates pro-inflammatory signaling pathways and dysregulates both cell proliferation and apoptotic mechanisms. These pathways contribute to systemic toxicity, increasing the risk of malignancies, cardiovascular diseases, and kidney dysfunction [31].

## MECHANISMS OF ARSENIC-INDUCED KIDNEY DAMAGE

Arsenic poses a significant threat to renal health due to the kidneys’ central role in filtering blood and excreting toxins. The nephrotoxic effects of arsenic involve multiple interconnected mechanisms:

### Oxidative stress and kidney injury

Arsenic metabolism generates ROS, initiating lipid peroxidation and compromising membrane integrity in renal tubular cells [32, 33]. This damages ion transport, inhibits antioxidant enzymes such as superoxide dismutase (SOD) and glutathione peroxidase (GPx), and results in DNA and protein oxidation, ultimately triggering apoptosis [34]. ROS also activate redox-sensitive transcription factors such as nuclear factor kappa (NF-κB) and activator protein 1 (AP-1), which upregulate inflammatory cytokines such as tumor necrosis factor alpha (TNF-α), interleukin-6 (IL-6), and interleukin-1 beta (IL-1β), thereby promoting renal inflammation and fibrosis [35].

### Mitochondrial dysfunction in arsenic toxicity

Arsenic disrupts mitochondrial homeostasis through activation of the nucleotide-binding domain, leucine-rich-containing family, pyrin domain-containing-3 (NLRP3)-transforming growth factor-beta 1 (TGF-β1)/Suppressors of Mothers Against Decapentaplegic (SMAD) signaling pathway, which promotes renal inflammation and fibrosis. It induces mitochondrial fragmentation and mitophagy, contributing to fibrogenic remodeling. The SIRT1–PINK1 pathway, which regulates mitophagy, plays a protective role by removing damaged mitochondria [36]. Inhibition of this pathway exacerbates nephrotoxicity and inflammation [37]. Arsenic also activates inflammatory signaling cascades, including NF-κB and p38 Mitogen-activated Protein Kinase (MAPK), further linking mitochondrial damage with renal injury [38, 39].

### Cell death pathways: Apoptosis and necrosis

Apoptosis is a key mode of arsenic-induced renal cytotoxicity. Arsenic exposure increases ROS levels, leading to mitochondrial dysfunction and cytochrome c release, which activates caspase-3 and initiates apoptosis [40, 41]. In addition, arsenic suppresses the Ak strain transforming (Akt) pathway, reducing anti-apoptotic proteins such as B-cell lymphoma 2 (Bcl-2) and increasing pro-apoptotic markers such as Bcl-2-associated X protein (Bax-1) [42]. This imbalance promotes programmed cell death and contributes to renal degeneration [43].

### Endoplasmic reticulum (ER) stress and renal dysfunction

Arsenic causes ER stress by promoting the accumulation of misfolded proteins, thereby triggering the unfolded protein response aimed at restoring ER function [44, 45]. However, persistent ER stress activates apoptotic pathways through protein kinase R (PKR)-like ER kinase, activating transcription factor 6, and inositol-requiring enzyme 1 (PERK, ATF6, and IRE1) signaling. PERK-induced expression of C/EBP homologous protein (CHOP) leads to apoptosis and calcium imbalance. Chronic ER stress has been linked to tubulointerstitial fibrosis and glomerular damage [46, 47].

### Epigenetic alterations in arsenic-induced nephrotoxi- city

Arsenic alters gene expression through epigenetic mechanisms, including DNA methylation, histone modification, and RNA methylation. Changes in DNA methyltransferase activity result in both hypermethylation and hypomethylation, disrupting gene regulation and impairing DNA repair processes. Histone acetylation and methylation also affect chromatin structure and transcription, promoting inflammatory gene expression such as IL-8 [48, 49].

Moreover, arsenic disrupts N6-methyladenosine (m6A) RNA methylation, which is crucial for gene expression and messenger RNA (mRNA) stability. The increased expression of methyltransferases, such as methyltransferase-like 3 (METTL3), suggests a role for m6A dysregulation in arsenic-induced renal injury and nephron developmental impairments [50, 51].

## FERROPTOSIS AND LIPID PEROXIDATION IN KIDNEY INJURY

Ferroptosis is a regulated, iron-dependent form of cell death marked by excessive accumulation of lipid peroxides. It plays a significant role in arsenic-induced renal injury by disrupting iron metabolism and weakening endogenous antioxidant defenses. Arsenic exposure leads to dysregulation of iron homeostasis, resulting in pathological iron accumulation in renal epithelial cells [52, 53]. Polyunsaturated fatty acid (PUFA)-enriched phospholipids, which are highly susceptible to oxidation, are central substrates in ferroptosis. These undergo peroxidation through both enzymatic mechanisms (e.g., lipoxygenase) and non-enzymatic iron-catalyzed Fenton reactions, generating phospholipid hydroperoxides and toxic lipid radicals. Enzymes such as Acyl-CoA synthetase long-chain family member 4 and lysophosphatidylcholine acyltransferase 3 (ACSL4 and LPCAT3) promote PUFA incorporation into phospholipids, thereby increasing ferroptotic vulnerability [54, 55].

Key molecular regulators of ferroptosis in renal cells include:

- GPX4, downregulated by arsenic, normally suppresses lipid peroxidation
- ACSL4, upregulated during arsenic exposure, enhances PUFA incorporation
- Solute Carrier Family 7, Member 11 (SLC7A11), inhibited by arsenic, maintains intracellular glutathione (GSH) levels
- Dual oxidase 1 (DUOX1), upregulated through hypoxia-inducible factor 2α (HIF-2α) signaling, increases oxidative stress [56, 57].

Arsenic also disrupts the Ras-GTPase-activating protein SH3 domain-binding protein 1, F-box and leucine-rich repeat protein 5, and iron-regulatory protein 2 (G3BP1-FBXL5-IRP2) axis, exacerbating iron accumulation and lipid peroxidation. Simultaneously, it activates ROS production and pro-inflammatory pathways such as p38 MAPK and NF-κB, while impairing apoptotic and autophagic responses through mitochondrial dysfunction and caspase-3 activation – all converging to amplify ferroptosis and kidney injury [58].

The HIF-2α/DUOX1/GPX4 signaling axis has emerged as a critical regulator of arsenic-induced ferroptosis. Structural mitochondrial damage – such as matrix condensation and cristae disintegration – is a hallmark of this process. Inflammatory cytokines further aggravate renal injury by amplifying oxidative stress and cellular damage.

*In vivo* and *in vitro* studies confirm that arsenic exposure triggers ferroptosis in renal tissues, marked by elevated ROS and iron accumulation. Ferroptosis inhibitors like ferrostatin-1 have shown protective effects by reducing lipid peroxidation. Similarly, Ferroptosis Suppressor Protein 1 mitigates ferroptosis through coenzyme Q10 (CoQ10) regeneration and suppression of lipid peroxide buildup [59, 60]. These findings underscore ferroptosis inhibition as a promising therapeutic strategy against arsenic-induced nephrotoxicity.

## DYSREGULATION OF AUTOPHAGY AND ITS IMPACT ON NEPHROTOXICITY

Autophagy is a fundamental catabolic process that degrades and recycles damaged organelles and macromolecules, thereby maintaining cellular homeostasis. It is which are essential for protecting renal cells from oxidative stress, nutrient deprivation, and xenobiotic toxicity. However, arsenic exposure impairs autophagic flux, exacerbating oxidative damage and promoting apoptosis – key contributors to acute kidney injury (AKI) and CKD.

Arsenic interferes with autophagosome-lysosome fusion by disrupting the STX17–SNAP29–VAMP8 SNARE complex, a process mediated by arsenic-induced O-GlcNAcylation of SNAP29. This impairs vesicular dynamics which are essential for autophagic clearance. Arsenic also activates the mammalian target of rapamycin pathway, a negative regulator of autophagy and a central controller of cell growth and metabolism.

The resulting autophagic dysfunction has diverse effects on renal cell types, including tubular epithelial cells, podocytes, and glomerular endothelial cells. In addition, arsenic compromises lysosomal biogenesis and destabilizes lysosomal membrane integrity, further reducing autophagic efficiency. This impaired clearance of cellular debris elevates oxidative stress and accelerates renal injury. Chronic arsenic exposure sustains these effects, promoting renal fibrosis and accelerating CKD progression [61, 62].

## CELLULAR SENESCENCE AND PROGRESSIVE RENAL DECLINE

Arsenic contributes significantly to renal cellular senescence and tissue aging by triggering a range of pathological processes. These include epigenetic alterations, telomere shortening, activation of the senescence-associated secretory phenotype (SASP), and mitochondrial dysfunction. Collectively, these changes result in irreversible cell cycle arrest, increased secretion of pro-inflammatory cytokines and matrix-degrading enzymes, amplifying inflammation and oxidative stress in renal tissue.

The accumulation of senescent cells in the kidney reduces tissue regenerative capacity and increases susceptibility to CKD and age-related nephropathies [63]. Arsenic-induced epithelial-mesenchymal transition further contributes to renal fibrosis by upregulating fibrotic mediators such as collagen I, fibronectin, and TGF-β [64].

**Figure 2:**
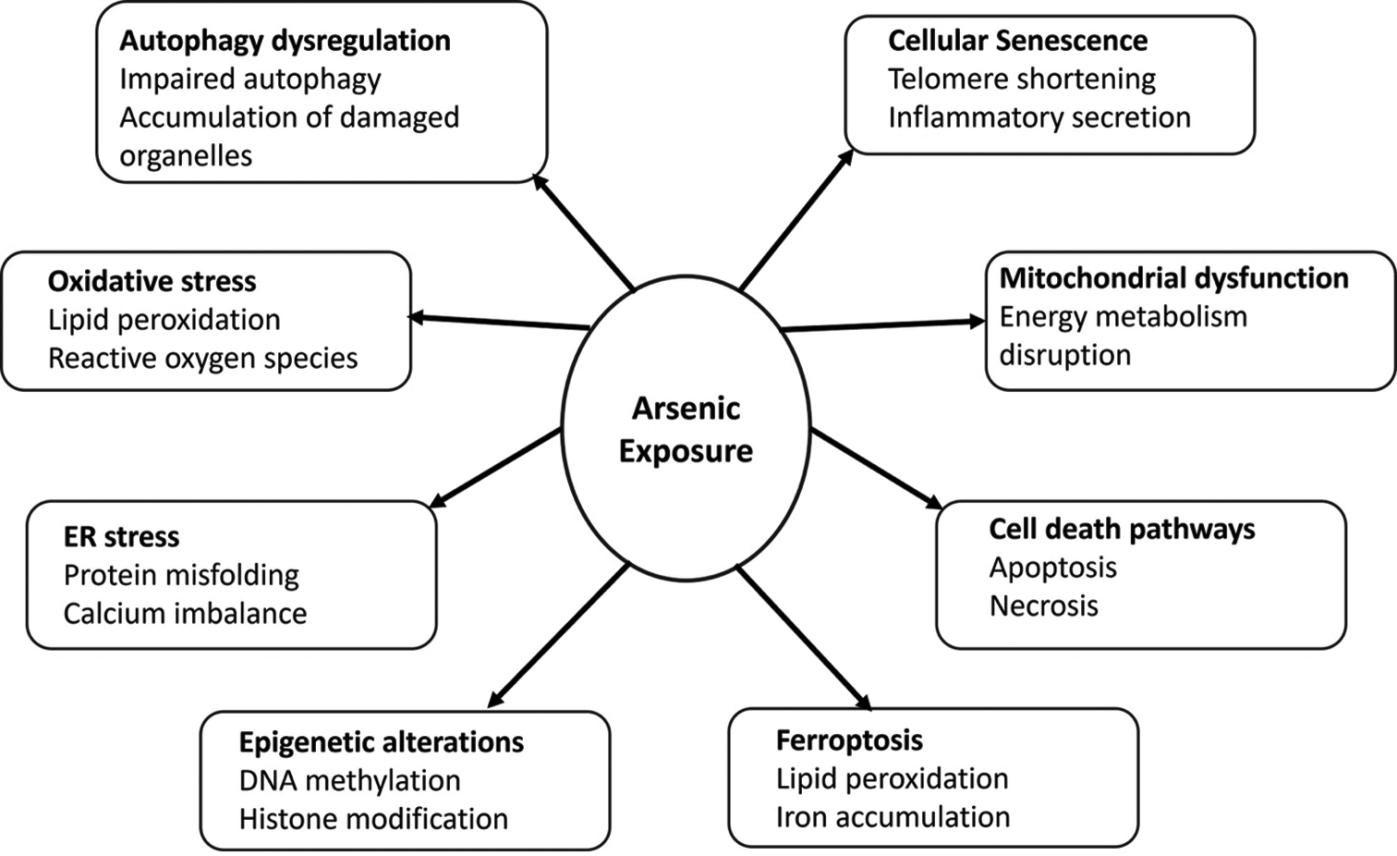
Mechanisms of arsenic-induced nephrotoxicity [65, 66].

In both renal tubular epithelial and endothelial cells, arsenic accelerates senescence through excessive ROS production and activation of senescence regulators including p53 and p16^INK4a. These signaling pathways not only impair cellular homeostasis but also promote the secretion of SASP components – pro-inflammatory cytokines, growth factors, and proteases – that exacerbate tissue injury and drive progressive kidney degeneration (Figure 2) [65, 66].

## SYSTEMIC IMPACTS OF ARSENIC-INDUCED GUT DYSBIOSIS ON RENAL FUNCTION AND KIDNEY–MICROBIOME INTERACTION

Arsenic exposure profoundly disrupts the gut microbiota, increasing the prevalence of pathogenic bacteria such as *Escherichia-Shigella* and *Klebsiella*, while reducing beneficial genera such as *Lactobacillus*. This microbial imbalance alters key metabolic pathways and elevates lipopolysaccharide production, leading to gut dysbiosis that adversely impacts renal function. Arsenic also compromises the integrity of the intestinal barrier by downregulating tight junction proteins, including Occludin, Zonula Occludens-1, and Mucin-2. This enhances gut permeability, allowing the translocation of inflammatory endotoxins into systemic circulation, which in turn triggers pro-inflammatory cytokine responses – particularly IL-6, IL-8, and TNF-α. These inflammatory mediators further aggravate gut dysbiosis, promote systemic inflammation, and facilitate increased arsenic absorption and accumulation in the kidneys, thereby exacerbating nephrotoxicity [67, 68].

The gut microbiota plays a central role in arsenic bioaccumulation and biotransformation, making specific bacterial taxa potential biomarkers for arsenic exposure and toxicity. Species such as *Lachnoclostridium, Erysipelatoclostridium, Blautia, Lactobacillus, Enterococcus*, and *Citrobacter* have been linked to arsenic metabolism and modulation of its toxicity and excretion [69–71].

In rodent models, arsenic exposure leads to compositional shifts in gut microbiota, favoring Gram-negative bacteria and compromising the intestinal barrier. This results in systemic inflammation, neurotoxicity, and behavioral abnormalities, characterized by altered neurotransmitter profiles and microglial activation [72]. Fecal microbiota transplantation studies reveal that arsenic-induced gut dysbiosis contributes not only to nephrotoxicity but also to hepatotoxicity, as shown by increased liver inflammation and elevated liver enzyme activity.

In addition, arsenic interferes with lipid and amino acid metabolism, including pathways involving fatty acids, phospholipids, sphingolipids, cholesterol, and tryptophan, thereby aggravating metabolic dysfunction. Gut dysbiosis increases arsenic retention and impairs its excretion, whereas bacterial genera such as *Lactobacillus* and *Blautia* appear to facilitate arsenic detoxification, highlighting the microbiome’s role in modulating arsenic toxicity [73, 74].

## GENETIC AND ENVIRONMENTAL DETERMINANTS OF ARSENIC NEPHROTOXICITY AND PERSONALIZED MEDICINE APPROACHES

Arsenic-induced nephrotoxicity is a multifactorial condition shaped by an interplay of genetic predisposition, environmental exposure, and lifestyle factors. Understanding these determinants is essential for identifying high-risk individuals and developing tailored interventions.

### Genetic determinants of arsenic nephrotoxicity

Genetic variability significantly affects arsenic metabolism and detoxification. The AS3MT gene encodes arsenic (+3 oxidation state) methyltransferase, the primary enzyme responsible for arsenic methylation. Single-nucleotide polymorphisms (SNPs) such as rs3740393 and rs11191439 influence enzymatic activity and alter arsenic metabolite profiles, thereby modulating susceptibility to arsenic toxicity [75].

Polymorphisms in genes such as formimidoyltransferase cyclodeaminase (*FTCD*), glutathione S-transferase omega 1 (*GSTO1*), and glutathione S-transferase mu 1 (*GSTM1*) also affect arsenic biotransformation. For example, FTCD polymorphisms may impair histidine degradation and methyl group availability, reducing arsenic methylation efficiency. Similarly, GSTO1 and GSTM1 variants influence glutathione-dependent detoxification pathways, affecting the distribution and elimination of arsenic species [76, 77].

### Ethnic and regional differences in susceptibility

Geographic and environmental factors significantly influence arsenic exposure and associated nephrotoxicity. Regions with endemic arsenic contamination – such as Bangladesh, India, and Taiwan – report higher incidences of arsenic-related kidney diseases [78]. In the United States, dietary habits (e.g., high rice and wine intake) elevate arsenic exposure, particularly in Chinese and Hispanic populations. Notably, indigenous populations in northern Argentina possess unique AS3MT polymorphisms that enhance methylation efficiency, reducing arsenic toxicity. These findings underscore the interaction between genetic makeup and environmental exposure [79].

Improving water quality and modifying dietary patterns are practical steps that can reduce arsenic exposure and its renal consequences, especially in high-risk regions.

### Genotype-based risk classification and policy implications

Genotype-based classification offers a personalized approach to identifying individuals at risk of arsenic-induced nephrotoxicity. For instance, individuals with the AS3MT rs9527 variant are considered slow methylators, which results in elevated monomethylarsonic acid (MMA) levels and heightened risk of systemic toxicity [80, 81]. The GSTM1-null genotype similarly impairs arsenic detoxification, increasing renal vulnerability.

Other relevant genetic variants include *GSTT1, GSTP1, GSTO1*, and *GSTO2*, which collectively impact glutathione-dependent conjugation. Reduced GST activity exacerbates oxidative stress and inflammation. In addition, deficiencies in the nuclear factor erythroid 2-related factor 2 (Nrf2) pathway, a key cellular antioxidant regulator, increase susceptibility to renal cell apoptosis and ferroptosis [82–84]. High-risk genotypes such as myeloperoxidase (*MPO*), *AA*, and purine nucleoside phosphorylas*e* (*PNP*) variants further compound the risk by promoting inflammation and disrupting metabolic processes.

Arsenic also induces epigenetic modifications, such as DNA methyltransferase upregulation, contributing to renal fibrosis. It disrupts iron regulation and alters metabolite profiles – including linoleic acid and bile acids – which intensify kidney damage. Epigenetic therapies targeting these molecular alterations offer promising avenues for protecting genetically susceptible individuals [85–87].

An effective public health response should integrate genetic screening, environmental surveillance, and nutritional interventions. Early identification of high-risk genotypes – especially slow methylators and GST-null variants – can facilitate preventive strategies. Strengthening arsenic monitoring in water sources and enhancing detoxification through targeted antioxidant support can reduce the global disease burden. Emerging epigenetic therapies also hold potential as personalized interventions for arsenic-exposed populations (Table 2) [88, 89].

**Table 2:** Genotype-based risk and public health interventions [88, 89].

Genetic marker	Risk factor	Mechanism	Public health action
AS3MT rs9527	Skin lesions and toxicity	Inefficient methylation	Dietary interventions
GSTM1-Null	Nephrotoxicity risk	Impaired detoxification	Monitoring water quality
GSTP1 Ile105Val	Bladder cancer	Oxidative stress	Genotype screening
GSTO1, GSTO2	Skin lesions	Metabolism efficiency	Regulations on water safety
MPO AA	Cancer risk	Inflammation pathway	Targeted therapies
Nrf2 Deficiency	Kidney damage	Antioxidant pathway	Antioxidant supplementation

GSTM1=Glutathione S-transferase mu 1, GSTO1=Glutathione S-transferase omega 1, GSTO2=Glutathione S-transferase omega 2, Nrf2=Nuclear factor erythroid 2-related factor 2, AS3MT=Arsenic (+3 oxidation state) methyltransferase, MPO AA=Myeloperoxidase Antibody, GSTP1=Glutathione S-transferase pi 1

### Personalized medicine approaches for arsenic exposure mitigation

Personalized medicine offers a promising avenue for mitigating the health risks associated with arsenic exposure by tailoring interventions to individual genetic, environmental, and behavioral profiles. Genetic screening plays a pivotal role in identifying individuals with impaired arsenic metabolism, who are at increased risk of toxicity and long-term health complications.

Therapeutic approaches such as chelation therapy, utilizing agents such as dimercaptosuccinic acid (DMSA) and monoisoamyl-DMSA (MiADMSA), have shown efficacy in enhancing arsenic excretion and reducing oxidative damage [90]. Nutritional interventions that support methylation pathways – particularly supplementation with vitamin B12 and folate – can improve arsenic detoxification. In addition, lifestyle modifications, including reduced consumption of arsenic-rich foods such as specific rice varieties and seafood, can be personalized based on individual exposure levels.

Routine surveillance using arsenic-specific biomarkers is essential for monitoring exposure and assessing therapeutic efficacy. Personalized tracking of arsenic burden enables timely modifications to treatment plans and lifestyle, thereby enhancing prevention and clinical management in at-risk populations [91].

## EXPERIMENTAL EVIDENCE

The nephrotoxic potential of arsenic has been extensively studied in animal models, providing critical insights into underlying molecular mechanisms and pathological outcomes. These findings have strong translational relevance to human health (Table 3) [92–103].

**Table 3:** Summary data used to describe the animal models used to analyze the arsenic-induced nephrotoxicity.

Animal model	Key results	Pathway involved	Reference
Mice	Elevation of serum creatinine and blood urea nitrogen levels. It reduces the levels of total thiol and increases renal malondialdehyde. Levels of TNF-α, nitric oxide, NF-κB, and phosphorylated NF-κB were enhanced.	Increase in the protein expression of phosphorylated NF-κB	[92]
Sprague-Dawley rats	Increased malondialdehyde levels and decreased antioxidant levels. Upregulation of NF-κB and IL-1β, TNF-α, IL-6, iNOS, COX-2, MAPK14, MAPK15, JNK. Autophagy through beclin-1 activation and apoptosis by increasing caspase-3 and Bax levels and decreasing Bcl-2 expression.	Stimulation of NF-κB factor. Activation of the MAPK pathway suppression of AKT2 and FOXO 1 expressions.	[93]
Wistar rats	Elevated concentrations of myeloperoxidase, nitric oxide, malondialdehyde, and protein carbonyl in the kidney tissue. Increased levels (IL-1β) and (TNF-α) in renal tissue	Downregulation of the PI3K/Akt/mTOR pathway	[94]
Kunming mice	Histopathological changes in the kidneys Elevated levels of nephrotoxic biochemical markers. Increased generation of ROS and decreased MMP	Autophagy and proptosis through the ROS pathway,	[95]
Wistar rats	Reduced expression of GSTO1 mRNA and protein. Reduced expression of Aqp3, Mrp1, Mrp4, and Mdr1b Increased urinary NGAL and FABP3 levels and decreased plasma Klotho levels	Alteration in gene expression of proteins (GSTO1 mRNA, Aqp3, Mrp1, Mrp4, and Mdr1b, Renal Klotho mRNA)	[96]
Rats	Increased inflammatory markers in renal tissue	Activation of NF-κB and inhibition of Nrf2 pathways	[97]
NMRI mice	Increased levels of kidney markers, oxidative stress, apoptosis, and inflammation in mouse kidney tissue Antioxidant enzymes, such as superoxide dismutase, catalase, and the amount of total thiol decreased	Stimulation of NF-κB factor, thus stimulating various inflammatory cytokines	[98]
Sprague-Dawley rats	Vacuole formation, nuclear condensation, the presence of polymorphonuclear leukocyte accumulation, and overabundance of collagen deposition within renal tissue were observed. Increase in the number of oxidative stress markers	Decline in eNOS expression	[99]
C57BL/6 mice	Renal tubular damage and impairment of mitochondrial function. Downregulated mRNA/protein expression of SIRT1 and PGC-1α and upregulated mRNA/protein expression of PINK1, Parkin, Beclin1, ATG5, and LC3B *in vivo* and *in vitro*	Reduced mRNA and protein levels of SIRT1 and increased mRNA and protein levels associated with mitophagy	[100]
NMRI mice	Increased levels of inflammatory markers, urea, and creatinine	Excessive TBARS and NO production contribute to the damage	[101]
Wistar rats	Elevated levels of urea, creatinine, and uric acid. Elevated KIM-1 and cystatin-C levels Elevated levels of TGF-β in kidney tissue upregulation of miRNA-181 expression	Apoptotic pathway stimulation LR-4 activation NLRP3 and caspase-1 activation.	[102]
Sprague -Dawley rats	Serum urea and creatinine activation. Increased levels of ROS, malonaldehyde, IL-1β, TNF-α, PC, LOOH Reduction in the levels of superoxide dismutase, catalase, glutathione, and TSH groups	I3K and AKT inhibition.	[103]

COX2: Cyclooxygenase-2, IL-6=Interleukin-6, IL-1β=Interleukin-1 beta, TNF-α=Tumor necrosis factor alpha, Nrf2=Nuclear factor erythroid 2-related factor 2, eNOS=Endothelial nitric oxide synthase, iNOS=Inducible nitric oxide synthase, NF-κB=Nuclear factor kappa B, mRNA=Messenger RNA, KIM-1=Kidney injury molecule-1, TGF-β=Transforming growth factor-beta 1, MiRNA=Micro RNA, PC=Protein carbonyls, LOOH=Lipid hydroperoxides, TSH=Total sulphydryl, GSTO1=Glutathione S-transferase omega 1, ROS=Reactive oxygen species, NGAL=Neutrophil gelatinase-associated lipocalin, MAPK=Mitogen-Activated Protein Kinase, JNK=c-Jun N-terminal kinase, Bax=Bcl-2-associated X protein, Bcl-2=B-cell lymphoma 2, AKT2=AKT serine/threonine kinase 2, FOXO 1=Forkhead box O, PI3K/Akt/mTOR=Phosphoinositide 3-kinase, Protein kinase B, Mammalian Target of Rapamycin MMP=Mitochondrial membrane potential, AQP3=Aquaporin-3 Mrp1=Multidrug resistance protein 1, Mrp4=Multidrug resistance protein 4 Mdr1b=Multidrug resistance protein 1b. SIRT1=silent mating type information regulation 2 homolog) 1, PINK1=PTEN-induced putative kinase, ATG5=autophagy-related protein 5, LC3B=Microtubule-associated protein 1 light chain 3 beta, PGC-1α= Peroxisome proliferator-activated receptor gamma coactivator 1-alpha, NMRI=nuclear magnetic resonance imaging

### Translation of experimental findings to clinical settings

Rodent models of arsenic-induced renal injury exhibit pathological and biochemical features similar to those observed in human cohorts. Elevated levels of serum creatinine, blood urea nitrogen, and uric acid have been reported in both experimental animals and exposed human populations [104]. Histopathological evidence, including renal tubular damage and fibrosis, has also been consistently documented across species.

Urinary biomarkers, such as β2M, N-acetyl-β-D-glucosaminidase (NAG), and malondialdehyde, correlate strongly with arsenic exposure and serve as non-invasive tools for the early detection of renal dysfunction. Furthermore, inflammatory mediators and microRNAs (miRNAs) (e.g., miR-191), which are elevated in exposed humans, mirror observations in rodent models [105].

Epidemiological studies confirm the association between chronic arsenic exposure and increased risk of CKD and renal fibrosis. Molecular pathways identified in animal studies, such as the Hippo-yes-associated protein 1 (YAP1)/HIF-1α axis, offer promising therapeutic targets for clinical application [106]. Co-exposure to arsenic and other nephrotoxicants like cadmium has been shown to exacerbate renal damage, further underscoring the translational value of these models [107].

## EARLY BIOMARKERS FOR DETECTING ARSENIC-INDUCED KIDNEY DAMAGE

Early diagnosis of arsenic-induced nephrotoxicity is critical for preventing irreversible damage. Several biomarkers, both conventional and novel, have demonstrated effectiveness in identifying early renal impairment.

### KIM-1

KIM-1 is a type I transmembrane glycoprotein that is markedly upregulated in proximal tubular epithelial cells following toxic insult [108]. It can be measured in urine and serum, and its levels correlate with the extent of tubular damage [109]. Immunohistochemical detection in renal biopsies also confirms its utility in assessing the severity of renal injury [110]. Notably, elevated KIM-1 levels have been associated with changes in serum creatinine, making it a valuable prognostic marker for predicting recovery [111].

### NGAL

NGAL is a 25-kDa protein secreted by activated neutrophils and injured renal tubular cells. Its levels rise rapidly in urine and plasma following acute kidney insult, offering an early indication of AKI [112]. NGAL has demonstrated high sensitivity and specificity in various clinical scenarios and meta-analyses, including neonatal asphyxia, supporting its role in early diagnosis and CKD management [113, 114].

### N-acetyl-β-D-glucosaminidase (NAG)

NAG is a lysosomal enzyme predominantly located in proximal tubule cells. Under normal conditions, it does not pass the glomerular barrier. Elevated urinary NAG activity reflects early tubular injury, serving as a reliable biomarker for both AKI and CKD, particularly in nephrotoxic drug-induced damage [115, 116].

### β2M

β2M is a low-molecular-weight protein filtered by the glomerulus and reabsorbed in the proximal tubules. Its elevated presence in urine or serum indicates proximal tubular dysfunction. Studies by Qiu *et al*. [117] and Chin *et al*. [118] in Taiwanese adults have demonstrated a significant association between urinary β2M levels and arsenic exposure, particularly to inorganic and arsenate species. β2M has proven useful in multi-metal exposure scenarios and is consistently elevated in arsenic-induced renal impairment [119, 120].

### α1-Microglobulin (α1-MG)

**Figure 3:**
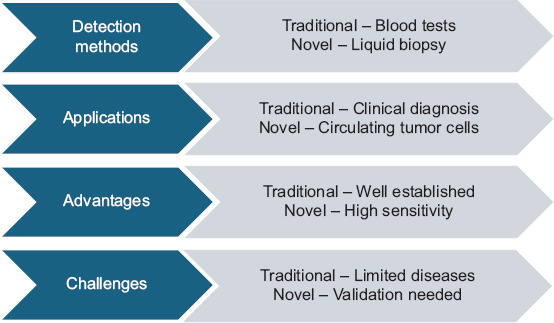
Comparison between traditional and novel biomarkers [121].

α1-MG is a liver-derived glycoprotein that is freely filtered by the glomeruli and reabsorbed by proximal tubules. Increased urinary levels of α1-MG signal early tubular dysfunction, especially from arsenic-induced oxidative and inflammatory damage. Owing to its sensitivity to subtle changes, α1-MG is gaining prominence as an early marker of arsenic-associated renal injury (Figure 3 and Table 4) [121].

**Table 4:** Comparison of biomarkers for kidney injury [121].

Biomarker	Source	Detection sample	Key functions	Clinical significance
KIM-1	Proximal tubule cells	Urine, Blood	Type 1 transmembrane protein upregulated post-injury	The early identification of AKI and CKD is associated with the severity of renal damage and is more effective than conventional markers.
NGAL	Neutrophils, Renal tubular cells	Urine, blood	Type 1 transmembrane protein upregulated post-injury	Rapid AKI detection and high sensitivity in critical care and post-surgery settings
NAG	Proximal tubule cell lysosomes	Urine	Enzymes indicating proximal tubular cell damage	Sensitive marker for nephrotoxic drug-induced kidney injury
β2-MG	Blood plasma protein levels	Urine	Small protein filtered by glomeruli and reabsorbed by the proximal tubules	Early detection of renal tubular dysfunction, useful in monitoring arsenic toxicity
α1-MG	Liver synthesis	Urine	Low-molecular-weight glycoprotein reflecting proximal tubular function	Sensitive to arsenic-induced nephrotoxicity, an early marker for tubular dysfunction

KIM-1=Kidney injury molecule-1, NGAL=Neutrophil gelatinase-associated lipocalin, β2MG=β2-microglobulinG, α1-MG: α1-Microglobulin, AKI=Acute kidney injury, CKD: Chronic kidney disease

## EMERGING AND UNCONVENTIONAL BIOMARKERS FOR ARSENIC-INDUCED NEPHROTOXICITY

Emerging technologies, such as miRNAs, metabolomics, and artificial intelligence (AI) are transforming the landscape of nephrotoxicity diagnostics. These approaches provide novel insights into arsenic-induced kidney injury and enhance diagnostic accuracy through the discovery of early biomarkers and the development of integrative predictive models.

### miRNAs as non-invasive biomarkers and therapeutic targets

miRNAs are small, non-coding RNAs that regulate gene expression and cellular processes at the post-transcriptional level. Due to their remarkable stability in plasma and urine, miRNAs are attractive candidates for non-invasive biomarkers. Changes in miRNA expression profiles reflect cellular responses to arsenic-induced oxidative stress, inflammation, and apoptosis. For instance, miR-21 and miR-155 are significantly upregulated following arsenic exposure and correlate with increased oxidative stress and pro-inflammatory signaling [122].

Urinary miRNAs such as miR-30a and miR-92a have also been identified in patients with AKI caused by nephrotoxins, highlighting their diagnostic potential for early-stage renal damage [123]. Notably, miR-21 directly regulates the NRF2 antioxidant pathway, underscoring its dual role as both a biomarker and a therapeutic target [124].

### Metabolomics for system-level insights

Metabolomic profiling reveals widespread alterations in renal metabolic pathways resulting from arsenic exposure. Disruptions in glycolysis, the tricarboxylic acid cycle, and amino acid metabolism have been consistently observed in individuals exposed to arsenic [125]. These metabolic impairments affect protein synthesis, redox balance, and membrane stability. Altered lipid metabolism contributes to phospholipid degradation, apoptosis, and compromised membrane integrity. Arsenic exposure also hampers glutathione biosynthesis, exacerbating oxidative stress in renal tissues [126].

Advanced techniques, such as mass spectrometry and principal component analysis, enable the identification of unique metabolic signatures. When integrated with transcriptomic and proteomic data, metabolomic analysis facilitates a comprehensive understanding of arsenic-induced nephrotoxicity and supports the identification of sensitive and specific biomarkers for early diagnosis.

## AI-DRIVEN BIOMARKER DISCOVERY AND MACHINE LEARNING (ML) IN NEPHROTOXICITY DIAGNOSTICS

AI, particularly ML, has emerged as a powerful tool in biomarker discovery and nephrotoxicity prediction. Predictive ML models have been successfully applied to forecast nephrotoxicity caused by agents such as colistin and vancomycin [127]. These models analyze large molecular datasets to identify key features and toxicity signatures.

In a notable study, 72 classification models were built using various molecular fingerprints and algorithms to predict nephrotoxic potential, demonstrating the feasibility of AI-based diagnostics [128]. ML has also been integrated into pharmaceutical development pipelines for preclinical screening of chemical libraries and herbal compounds, enabling early detection of nephrotoxic risk [129].

## COMPOSITE BIOMARKER PANELS FOR KIDNEY DISEASE DIAGNOSIS

Composite biomarker panels and algorithm-based diagnostic tools offer enhanced sensitivity and specificity compared to traditional single-marker assays. These tools represent a major advancement in the early detection and staging of CKD and AKI.

- The KidneyIntelX™ platform utilizes plasma levels of KIM-1, soluble tumor necrosis factor (sTNF) receptor-1, and sTNF receptor-2 to predict diabetic kidney disease progression over 5 years [130]
- A biomarker panel comprising serum creatinine, osteopontin, tryptase, urea, and estimated glomerular filtration rate (eGFR) achieved an 84.3% accuracy in predicting CKD progression [131]
- Combining standard markers such as creatinine and cystatin C with sensitive biomarkers such as beta-trace protein (BTP), tissue inhibitor of metalloproteinase-1, TGF-β, asymmetric dimethylarginine (ADMA), TNF-α, and N-terminal pro-B-type natriuretic peptide (proBNP) have shown improved diagnostic performance for early CKD detection [132]
- Panels integrating tumor necrosis factor receptor (TNFR)-1, TNFR-2, and KIM-1 have demonstrated superior predictive accuracy for renal function decline
- The kidney injury test uses urinary markers – including cell-free DNA (cfDNA), methylated cfDNA, clusterin, C-X-C motif chemokine ligand 10 (CXCL10), total protein, and creatinine – to generate a diagnostic score with high sensitivity and specificity, even when eGFR and proteinuria remain within normal ranges
- For AKI detection, urinary biomarker panels combining NGAL, KIM-1, cystatin C, and hemojuvelin have shown strong predictive value. In perioperative and critical care settings, the combination of NGAL, KIM-1, and cystatin C in plasma or urine enhances diagnostic precision
- Additional composite panels that include albumin, β2M, clusterin, osteopontin, and BTP have been validated in various clinical contexts [133, 134].

**Figure 4:**
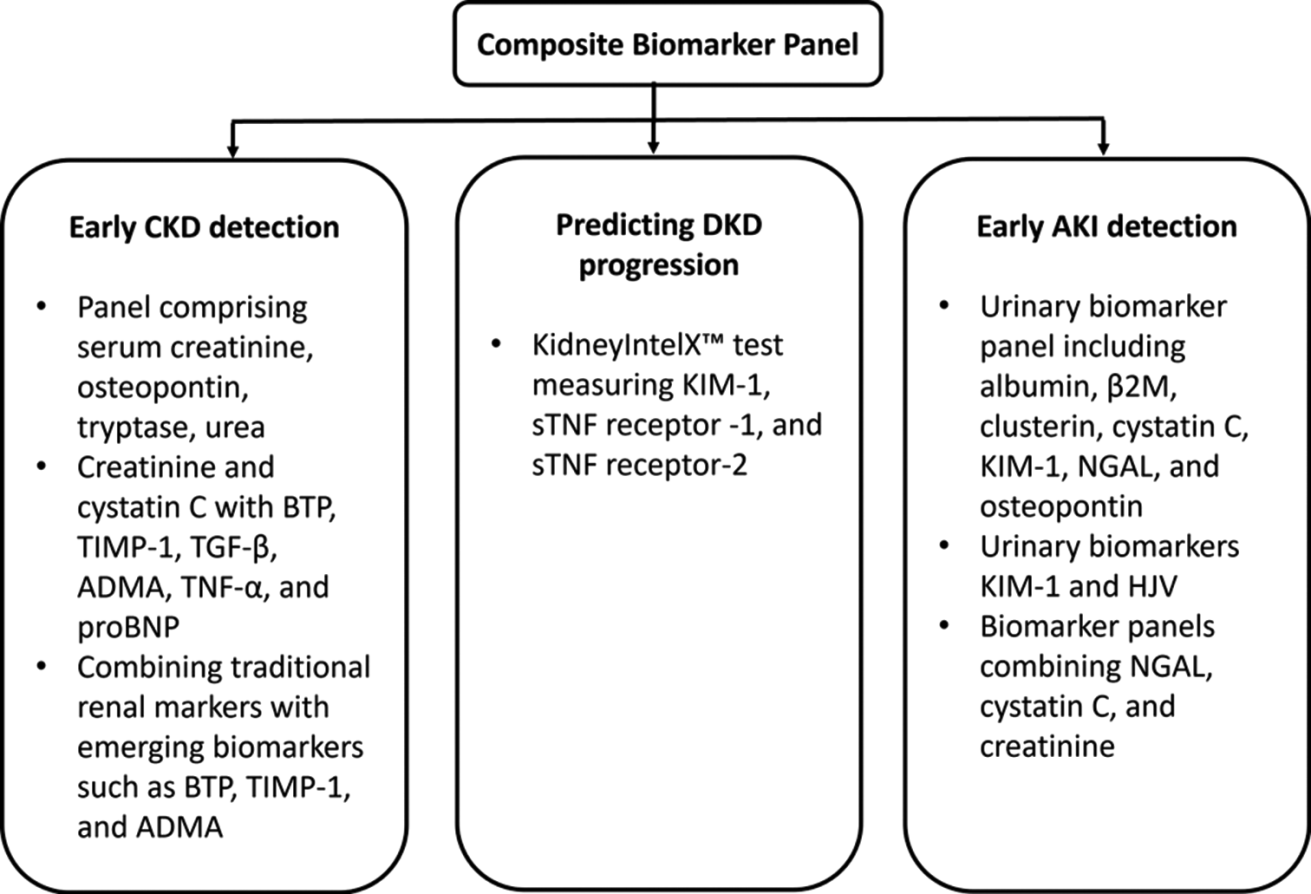
Composite biomarker panels for the diagnosis of kidney disease [133, 134].

These advanced panels not only enhance early detection but also enable risk stratification and tailored therapeutic interventions, marking a shift toward precision nephrology (Figure 4) [133, 134].

## PREVENTIVE, THERAPEUTIC, AND DETOXIFICATION STRATEGIES FOR KIDNEY PROTECTION

### Preventive strategies

An integrated approach involving public health policy, environmental monitoring, genetic screening, and nutritional modulation is critical to preventing arsenic-induced nephrotoxicity.

#### Reducing arsenic exposure

Eliminating arsenic from drinking water remains the cornerstone of public health efforts. Advanced water filtration technologies, such as reverse osmosis (RO) and activated alumina filters, can remove up to 99% of arsenic, making them highly effective in areas endemic to arsenic [135]. RO systems are widely recommended due to their superior removal capacity, while the efficacy of activated alumina filters is influenced by the pH of the water. Implementing these technologies in areas contaminated with arsenic is essential for reducing population-level exposure and subsequent kidney damage.

#### Genotyping of at-risk populations

Genetic screening of individuals in arsenic-endemic areas – such as Bangladesh and India – is valuable for identifying populations with increased genetic susceptibility. Variants in arsenic (+3 oxidation state) methyltransferase (AS3MT), glutathione S-transferase Mu 1 (GSTM1), and other detoxification-related genes influence arsenic metabolism and toxicity. Genotyping enables targeted risk assessment, personalized health guidance, and optimized resource allocation [136]. The success of such initiatives depends on local health infrastructure, community participation, and support from global partnerships [137].

#### Biosensors for real-time arsenic detection

Next-generation biosensors offer cost-effective and rapid detection of arsenic in water sources. These include nanomaterial-based sensors, whole-cell biosensors, and portable electrochemical devices, making them suitable for both urban and rural deployment [138, 139]. Integration of biosensors into water safety protocols enhances community awareness, facilitates immediate remediation, and supports ongoing surveillance programs.

#### AI-based surveillance and environmental monitoring

AI and Internet of Things platforms are increasingly used to monitor arsenic contamination by analyzing data from sensors, health records, and geospatial models. AI systems can predict exposure hotspots, guide remediation efforts, and optimize health responses [140]. Incorporating AI, biosensors, and genotyping into traditional mitigation frameworks enables a dynamic and adaptive strategy for arsenic management and protection of kidney health.

## NUTRITIONAL AND LIFESTYLE MODIFICATIONS

Nutritional and behavioral changes are a key to both preventing and alleviating arsenic-induced renal toxicity.

- A low-fat diet may decrease the risk of arsenic-related kidney damage [141]
- Antioxidant-rich foods such as berries, citrus fruits, spinach, and nuts enhance cellular defense mechanisms and neutralize ROS [142]
- Adequate hydration supports arsenic excretion and kidney detoxification [143]
- Avoiding arsenic-contaminated foods, including rice and groundwater from endemic regions, is crucial for reducing dietary exposure
- Nutraceuticals and functional foods may offer protective benefits without adverse effects
- Regular physical activity improves metabolic efficiency and detoxification [144]
- Avoidance of tobacco and alcohol, both of which exacerbate renal impairment, is strongly advised
- Stress reduction techniques, such as yoga and meditation, contribute to overall kidney health by modulating chronic inflammation
- Routine medical checkups are crucial for the early detection of nephrotoxicity and for enabling timely intervention [145].

### Therapeutic strategies

#### Antioxidant therapy

Antioxidants play a central role in neutralizing arsenic-induced ROS and reducing renal inflammation and damage.

- Vitamin C is a potent ROS scavenger that boosts endogenous antioxidant enzyme activity. It mitigates renal oxidative damage and inflammation [146]
- Vitamin E, a lipid-soluble antioxidant, stabilizes cellular membranes and reduces oxidative stress in renal tissues. Clinical evidence supports its role in improving kidney function under arsenic exposure [147]
- N-Acetylcysteine replenishes glutathione levels, enhancing intracellular redox balance and reducing oxidative burden in kidney cells [148]
- Alpha-lipoic acid is another potent antioxidant that enhances renal function by reducing oxidative stress and inflammatory signaling in renal tissues [149].

These antioxidant agents represent an accessible, cost-effective means of attenuating arsenic toxicity and preserving kidney function, particularly when integrated into comprehensive detoxification and dietary strategies.

## HERBAL REMEDIES

### Herbal and natural compounds for arsenic-induced kidney protection

Curcumin, the primary active ingredient in turmeric, possesses potent antioxidant and anti-inflammatory characteristics. It reduces oxidative stress and inflammation in renal tissues, thereby protecting against arsenic-induced nephrotoxicity. Curcumin upregulates endogenous antioxidant systems, whereas resveratrol exerts protective effects throughthe activation of silent mating type information regulation 2 homolog 1 (SIRT1) and mitochondrial preservation. Curcumin modulates various signaling pathways, including the Nrf2 pathway, which enhances the expression of antioxidant proteins and enzymes [150]. Curcumin alleviated arsenic-induced renal damage in rats by downregulating the expression of proinflammatory cytokines, such as TNF-α and IL-6. Curcumin also enhances the actions of SOD and catalase, which contribute to a decrease in oxidative damage within renal tissues [151, 152].

Tetramethylpyrazine (TMP) mitigates arsenic-induced renal damage through several key mechanisms. First, it mitigates oxidative stress by lowering ROS production and increasing antioxidant levels, such as glutathione. It also protects mitochondrial function by preserving cytochrome c oxidase activity and mitochondrial membrane potential. In addition, TMP exhibits anti-inflammatory effects by inhibiting pro-inflammatory pathways, such as NF-κB and p38 MAPK, thereby reducing the expression of inflammatory markers, including cyclooxygenase-2 and TNF-α. TMP also prevents cell death by reducing apoptosis and regulating autophagic flux through modulation of the Yes-associated protein 1/nuclear factor erythroid 2-related factor 2/ubiquitin-binding protein (YAP1–Nrf2–p62) pathway. This regulation helps prevent the accumulation of damaged proteins and promotes cell survival [153, 154].

Tannic acid protects the kidneys against arsenic-induced damage by mitigating oxidative stress, inflammation, and cell death. It accomplishes this by influencing critical biological pathways, notably by downregulating the expression of NF-κB while upregulating Nrf2 and kelch-like ECH-associated protein 1 (Keap1). These alterations contribute to a reduction in nephritic, oxidative stress, and inflammatory markers within renal tissue, thereby offering protection against renal damage [155].

Astaxanthin alleviates the renal damage caused by arsenic exposure due to its strong antioxidant and anti-inflammatory effects. It reduces oxidative stress by counteracting free radicals and improving the activity of antioxidant enzymes, including SOD and catalase. This helps to lower malondialdehyde levels, thereby protecting cellular membranes from damage. Astaxanthin also reduces inflammation by lowering the levels of TNF-α and IL-6, which are crucial for alleviating renal inflammation. The administration of astaxanthin has been shown to reduce the levels of apoptotic markers, such as caspase-3, which in turn decreases cell death in renal tissues [156, 157].

**Figure 5:**
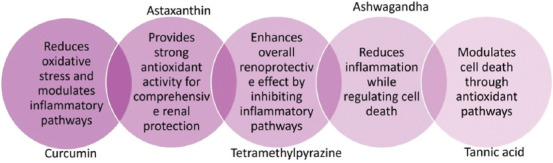
Natural compounds for renoprotection [158, 159].

Ashwagandha, an adaptogenic herb, has been shown to lower oxidative stress levels and enhance kidney function in cases of arsenic exposure. It enhances the body’s antioxidant defense mechanisms and reduces the levels of pro-inflammatory cytokines, such as TNF-α and IL-6, which are elevated in arsenic-induced renal damage, thereby safeguarding renal tissues from damage (Figure 5) [158, 159].

## NEXT-GENERATION THERAPIES FOR ARSENIC DETOXIFICATION

Traditional treatments, including chelation therapy and antioxidants, have demonstrated limited effectiveness in addressing arsenic-induced nephrotoxicity, highlighting the necessity for more sophisticated therapeutic options. Recent advancements in gene therapy, Clustered Regularly Interspaced Short Palindromic Repeats (CRISPR)-based interventions, nanotechnology, and microbiome modulation offer promising new avenues for arsenic detoxification, potentially improving the efficacy and specificity of treatment options.

### Gene therapy and CRISPR-based approaches

Gene therapy and CRISPR-based techniques offer novel methods by targeting essential genetic pathways related to detoxification, oxidative stress response, and cellular repair. Arsenic exposure primarily causes nephrotoxicity through mechanisms such as oxidative stress, inflammation, and apoptosis, with significant pathways, including MAPKs, NF-κB, and Nrf2, being crucial [160]. Traditional treatments often fail to address these underlying molecular mechanisms, necessitating the use of innovative genetic approaches. Gene therapy aims to correct or replace faulty genes, enhancing protective mechanisms, such as antioxidant defenses. For example, enhancing the expression of heme oxygenase-1 has been shown to counteract oxidative stress and inflammation, thereby promoting kidney protection [161].

On the other hand, CRISPR technology serves as a powerful and precise gene-editing tool, allowing researchers to target specific genes implicated in arsenic toxicity. The CRISPR/Cas9 system facilitates accurate genome modifications, making it an essential resource for investigating and potentially treating kidney diseases by directly addressing genetic mutations associated with arsenic exposure [162, 163]. Genome-wide CRISPR screening has identified genes, such as E2F1 that contributes to apoptosis through the p53 signaling pathway, providing a potential target for intervention [164]. CRISPR can be used to create *in vivo* models to better understand the impact of arsenic on kidney function and test therapeutic strategies [165]. Despite its promise, efficiently delivering CRISPR components to kidney cells while minimizing off-target effects remains a significant challenge. Advancements in delivery techniques and more precise gene-editing methods, such as prime and base editing, are essential for the successful application of CRISPR-based therapies in arsenic-induced nephrotoxicity [163].

### Nanotechnology for targeted detoxification

Nanotechnology offers a highly promising method for efficient and targeted detoxification, utilizing nanomaterials to enhance the removal of arsenic and protect the kidneys. Polymeric nanoparticles, when combined with chelating agents like MiADMSA, improve arsenic removal by enhancing the chelation process. In addition, they help reverse oxidative stress and histopathological changes in tissues, thereby improving kidney function. Selenium nanoparticles (SeNPs) have shown encouraging nephroprotective effects by reducing renal tissue fibrosis, inflammation, oxidative damage, and apoptosis. By enhancing the body’s natural detoxification mechanisms, SeNPs contribute to alleviating the toxic effects of arsenic on the kidneys [166]. The targeted delivery capability of nanotechnology further enhances the effectiveness of these interventions, allowing the concentration of therapeutic agents in the affected areas, thus improving treatment efficacy while minimizing side effects. Such integrative therapies effectively lower the arsenic burden while preserving renal structure and function.

### The role of the gut microbiome in arsenic detoxification

**Figure 6:**
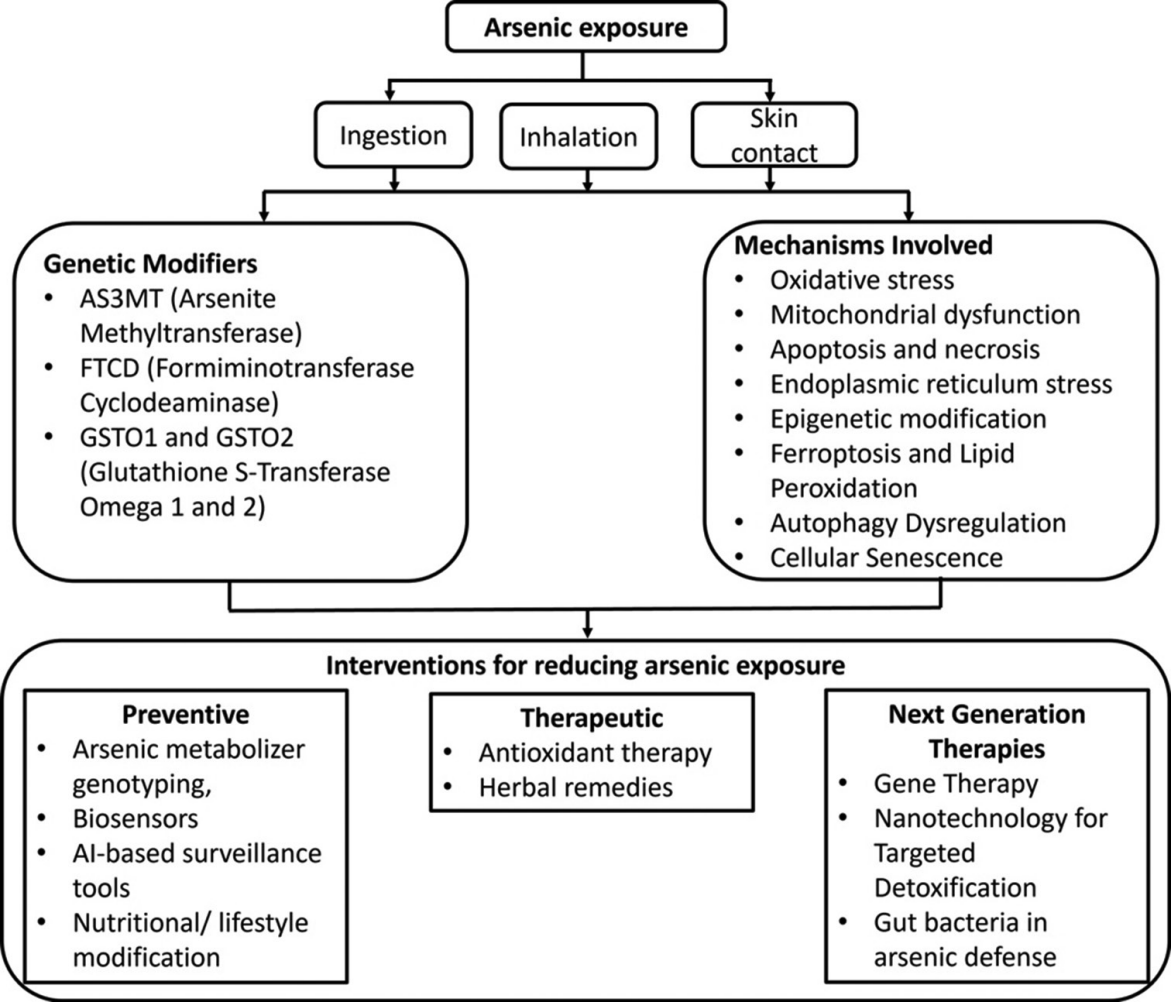
Holistic framework linking exposure, genetics, cell death pathways, and interventions [167, 168].

The modulation of the gut microbiome, which plays a crucial role in the biotransformation and accumulation of arsenic, is an emerging area of research in arsenic detoxification. Research indicates that a robust gut microbiome can enhance the elimination of arsenic through feces and reduce its accumulation in body tissues, such as the liver, kidneys, and brain. Studies have shown that mice with a complete gut microbiota have significantly lower arsenic concentrations in their tissues than mice lacking a microbiome, which exhibit more severe pathological changes. The presence of certain gut bacteria, including *Lactobacillus* and *Blautia*, has been linked to reduced arsenic bioaccumulation and increased biotransformation, highlighting the protective role of these microbes. Dietary modifications, probiotics, and prebiotics can help modulate gut microbiomes to decrease the absorption of arsenic and mitigate its harmful effects [167, 168]. This strategy offers a complementary therapeutic approach to support traditional methods of arsenic detoxification and prevent arsenic-induced diseases (Figure 6) [167, 168].

## UNDER-RESEARCHED REGIONS

Arsenic exposure remains a significant global public health threat, affecting both environmental and human health. More than 200 million people worldwide are at risk, with the highest burdens observed in South and Southeast Asia, particularly in Bangladesh and India, as well as in regions of Latin America, Africa, and Europe. Elevated levels of arsenic have been reported in Sub-Saharan Africa, including Limpopo, South Africa, where recent studies have identified contamination in water, soil, and human blood samples [169, 170].

Groundwater contamination has also been identified in Ghana and Nigeria, although there are limited comprehensive data. In Southeast Asia, Myanmar faces arsenic-related health risks, including oxidative DNA damage, but research on this subject is still sparse [171]. Arsenic contamination has been reported in 14 of 20 countries in South America, including Argentina, Bolivia, Brazil, Chile, and Peru. Socioeconomic factors, such as education level, influence arsenic exposure and health outcomes, especially in Europe [172]. While European populations face some dietary exposure, better diagnostic systems are needed to mitigate health risks [173, 174].

Low- and middle-income countries face the greatest challenges due to high levels of arsenic exposure and inadequate healthcare infrastructure. The densely populated regions of Asia and Latin America represent the highest public health risk from arsenic due to the convergence of high exposure and limited resources [175, 176] (Table 5).

**Table 5:** Regions with hidden arsenic burden and low diagnostic infrastructure [175, 176].

Region/Country	Main mode (s) of exposure	Key sources	Infrastructure/Context
South Asia (Bangladesh, India, Nepal)	Drinking water (groundwater)	Contaminated tube wells and irrigation	Widespread use of untreated groundwater, limited water testing, and health monitoring
Latin America (Argentina, Chile, Mexico, Uruguay)	Drinking water and food (rice, vegetables)	Natural geogenic sources, mining, and agriculture	Chronic exposure, limited biomonitoring, and limited public health resources
sub-Saharan Africa (South Africa, Ghana)	Drinking water, soil ingestion, and food	Borehole water, contaminated soil, and crops	Reliance on boreholes, minimal water quality surveillance, and low diagnostic access
Southeast Asia (Vietnam, Cambodia)	Drinking water and food	Groundwater and rice irrigation	High groundwater arsenic content, limited water treatment, and low diagnostic capacity
Europe	Food (dietary intake)	Rice, cereals, and seafood	Lower water exposure, but significant dietary sources; variable infrastructure

## UNRESOLVED QUESTIONS AND RESEARCH DIRECTIONS

Arsenic-induced nephrotoxicity is a multifaceted pathological condition involving oxidative stress, inflammatory cascades, apoptotic signaling, autophagic disruption, and cellular senescence. Although the mechanisms of arsenic toxicity are well understood, new molecular pathways are still being investigated. One notable pathway involves epigenetic changes, as arsenic exposure has been found to modify DNA methylation and histone alterations, subsequently affecting gene expression.

Furthermore, arsenic exposure triggers ferroptosis, a type of cell death characterized by lipid peroxidation and iron buildup, facilitated by the HIF-2α/DUOX1/GPX4 pathway. Another area of concern is arsenic’s interference with autophagy, a cellular process responsible for eliminating damaged components, which contributes to cell death and kidney dysfunction [177].

The role of miR-191 in modulating inflammatory responses and kidney dysfunction is a promising area of research, which opens new possibilities for exploration [178]. Although the Nrf2 antioxidant pathway is known to offer protective effects, the specific regulatory mechanisms involved in arsenic-induced nephrotoxicity are not yet fully understood [179].

Exposure to arsenic increases the levels of DNA methyltransferases (DNMTs), specifically DNMT3a and DNMT3b, which contribute to renal fibrosis [180]. Furthermore, arsenic exposure leads to changes in histone modification, including an increase in histone H3 trimethylation at lysine 36. This specific modification is associated with DNA damage and genomic instability.

Another important discovery is the alteration of IL-8 expression due to changes in DNA methylation and histone acetylation. These epigenetic modifications contribute to renal toxicity and heightened cell proliferation, thereby intensifying the harmful effects of arsenic on kidney function.

In addition, arsenic interacts with environmental toxins such as copper, pesticides, and antimony, worsening oxidative stress, apoptosis, and proptosis in kidney cells. The simultaneous presence of arsenic and copper activates the Nrf2 antioxidant pathway, leading to increased oxidative stress and a more pronounced proptosis and apoptosis of kidney cells. Similarly, the combination of arsenic and antimony contributes to oxidative stress, autophagy, and proptosis, and further worsens kidney damage.

Future investigations should aim to optimize current therapeutic modalities and uncover novel molecular targets for intervention [179]. Elucidating the interplay of these determinants is vital for designing targeted preventive measures and individualized therapeutic strategies to mitigate the harmful effects of arsenic exposure on kidney function.

## CONCLUSION

This comprehensive review elucidates the multifactorial nature of arsenic-induced nephrotoxicity, emphasizing the involvement of oxidative stress, mitochondrial dysfunction, ferroptosis, inflammation, apoptosis, autophagy disruption, cellular senescence, and epigenetic alterations. It further highlights the emerging roles of miRNAs, metabolomics, and AI-driven tools in advancing diagnostic precision and understanding molecular mechanisms. A range of preventive, therapeutic, and detoxification strategies – including antioxidant therapy, nutraceuticals such as curcumin and astaxanthin, gene-editing technologies, nanomedicine, and gut microbiome modulation – demonstrate considerable promise in mitigating arsenic toxicity.

The integration of advanced water filtration systems, biosensors, and AI surveillance into public health infrastructures is critical for real-time arsenic monitoring, especially in under-researched, high-risk regions. Personalized interventions, including genetic screening, tailored nutrition, and biomarker-guided monitoring, can improve early detection and risk stratification, reducing the burden of kidney disease. Novel therapeutics such as selenium nanoparticles, CRISPR-based gene editing, and microbiome-targeted therapies offer next-generation approaches to treatment.

This review provides an interdisciplinary synthesis bridging toxicology, nephrology, genomics, bioinformatics, and public health. It highlights not only well-established mechanisms but also emerging research trends and underexplored geographies. The inclusion of early biomarkers, therapeutic targets, and technological innovations enhances the translational relevance of the findings.

Despite significant progress, the review identifies key knowledge gaps, particularly in the clinical validation of biomarkers, long-term human studies, and regional exposure assessments in low-resource settings. Much of the mechanistic evidence is derived from animal models, requiring further translation to human populations. Moreover, the safe and effective deployment of advanced technologies, such as CRISPR, in kidney-specific contexts remains a challenge.

Future research should prioritize large-scale epidemiological studies in underrepresented regions, longitudinal validation of composite biomarker panels, exploration of epigenetic therapies and RNA-based drugs, and the development of safe delivery mechanisms for gene and nano-therapies. Integrating exposomics and multi-omic data will also be essential for advancing personalized medicine. Multidisciplinary collaborations will be necessary to accelerate these advances and translate bench discoveries into public health solutions.

Arsenic-induced nephrotoxicity represents a complex yet addressable global health issue. With a deeper understanding of its mechanistic underpinnings and the deployment of innovative diagnostic and therapeutic tools, there is a tangible opportunity to protect vulnerable populations, reduce renal disease burden, and build resilient health systems against environmental toxicants. Continued investment in research, surveillance, and targeted interventions will be pivotal to advancing kidney health in arsenic-affected regions worldwide.

## AUTHORS’ CONTRIBUTIONS

PLC and JP: Drafted the manuscript. PLC and JP: Revised and edited the manuscript. Both authors have read and approved the final manuscript.

## References

[1] Mandal B.K (2023). Changing concept of arsenic toxicity with development of speciation techniques. In:Handbook of Arsenic Toxicology.

[2] Adeloju S.B, Khan S, Patti A.F (2021). Arsenic contamination of groundwater and its implications for drinking water quality and human health in under-developed countries and remote communities-a review. Appl. Sci.

[3] Mahanta C, Choudhury R, Basu S, Hemani R, Dutta A, Barua P.P, Borah P.J, Bhattacharya M, Bhattacharya K, Alam W, Saikia L (2015). Preliminary assessment of arsenic distribution in Brahmaputra River basin of India based on examination of 56,180 public groundwater wells. In:Safe and Sustainable Use of Arsenic-Contaminated Aquifers in the Gangetic Plain:A Multidisciplinary Approach.

[4] Patterson R.M, Trouba K.J, Germolec D.R (2006). Immunotoxicology and inflammatory mechanisms of arsenic. In:Immunotoxicology and Immunopharmacology.

[5] Raessler M (2018). The arsenic contamination of drinking and groundwaters in Bangladesh:Featuring biogeochemical aspects and implications on public health. Arch. Environ. Contam. Toxicol.

[6] Polya D.A, Lawson M (2015). Geogenic and anthropogenic arsenic hazard in groundwaters and soils:In:Distribution, Nature Origin and Human Exposure Routes. Arsenic:Exposure Sources. Health Risks and Mechanisms of Toxicity.

[7] Palma-Lara I, Martínez-Castillo M, Quintana-Pérez J.C, Arellano-Mendoza M.G, Tamay-Cach F, Valenzuela-Limón O.L, García-Montalvo E.A, Hernández-Zavala A (2020). Arsenic exposure:A public health problem leading to several cancers. Regul. Toxicol. Pharmacol.

[8] Fatoki J.O, Badmus J.A (2022). Arsenic as an environmental and human health antagonist:A review of its toxicity and disease initiation. J. Hazard. Mater. Adv.

[9] Saxena A.K, Kumar A, Saxena A.K, Kumar A (2020). Effect of arsenic exposure in reproductive health. In:Fish Analysis for Drug and Chemicals Mediated Cellular Toxicity.

[10] Rodríguez R, Garcia-Gonzalez H, Hernández Z, Sanmiquel L (2024). Tackling arsenic and mercury contamination:Implications for sustainable mining and occupational health risks. Sustainability.

[11] Chen Y, Peng C, Zhang H, Cai Y, Yuan R, Song P, Zhang C, Yan Y (2023). Exposure to occupational risk factors is associated with the severity and progression of chronic obstructive pulmonary disease. Medicine (Baltimore).

[12] Pál L, Jenei T, McKee M, Kovács N, Vargha M, Bufa-Dőrr Z, Muhollari T, Bujdosó M.O, Sándor J, Szűcs S (2022). Health and economic gain attributable to the introduction of the World Health Organization's drinking water standard on arsenic level in Hungary:A nationwide retrospective study on cancer occurrence and ischemic heart disease mortality. Sci. Total Environ.

[13] Kim K.W, Chanpiwat P, Hanh H.T, Phan K, Sthiannopkao S (2011). Arsenic geochemistry of groundwater in Southeast Asia. Front. Med.

[14] Barla A, Sathyavelu S, Afsal F, Ojha M, Bose S (2018). Agronomics management for arsenic stress mitigation. In:Mechanisms of Arsenic Toxicity and Tolerance in Plants.

[15] Wang J, Wan Y, Cheng L, Xia W, Li Y, Xu S (2020). Arsenic in outdoor air particulate matter in China:Tiered study and implications for human exposure potential. Atmos. Pollut. Res.

[16] Rahaman M.S, Rahman M.M, Mise N, Sikder M.T, Ichihara G, Uddin M.K, Kurasaki M, Ichihara S (2021). Environmental arsenic exposure and its contribution to human diseases, toxicity mechanism and management. Environ. Pollut.

[17] Kenyon E.M, Hughes M.F, Adair B.M, Highfill J.H, Crecelius E.A, Clewell H.J, Yager J.W (2008). Tissue distribution and urinary excretion of inorganic arsenic and its methylated metabolites in C57BL6 mice following subchronic exposure to arsenate in drinking water. Toxicol. Appl. Pharmacol.

[18] Palma-Lara I, Martínez-Castillo M, Quintana-Pérez J.C, Arellano-Mendoza M.G, Tamay-Cach F, Valenzuela-Limón O.L, García-Montalvo E.A, Hernández-Zavala A (2020). Arsenic exposure:A public health problem leading to several cancers. Regul. Toxicol. Pharmacol.

[19] Chakraborti D, Singh S.K, Rashid M.H, Rahman M.M (2011). Arsenic:Occurrence in groundwater. Encyclopedia Environ. Health.

[20] Rahaman M.S, Mise N, Ichihara S (2022). Arsenic contamination in food chain in Bangladesh:A review on health hazards, socioeconomic impacts and implications. Hyg. Environ. Health Adv.

[21] Baghery F, Lau L.D.W, Mohamadi M, Vazirinejad R, Ahmadi Z, Javedani H, Eslami H, Nazari A (2023). Risk of urinary tract cancers following arsenic exposure and tobacco smoking:A review. Environ. Geochem. Health.

[22] Haschek W.M, Rousseaux C.G, Wallig M.A, Bolon B (2021). Haschek and Rousseaux's Handbook of Toxicologic Pathology. Vol. 1 and 47. Principles and Practice of Toxicologic Pathology.

[23] Khan M.A, Ho Y.S (2011). Arsenic in drinking water:A review on toxicological effects, mechanism of accumulation and remediation. Asian J. Chem.

[24] Dodmane P.R, Arnold L.L, Kakiuchi-Kiyota S, Qiu F, Liu X, Rennard S.I, Cohen S.M (2013). Cytotoxicity and gene expression changes induced by inorganic and organic trivalent arsenicals in human cells. Toxicology.

[25] Vergara-Gerónimo C.A, León Del Río A, Rodríguez-Dorantes M, Ostrosky-Wegman P, Salazar A.M (2021). Arsenic-protein interactions as a mechanism of arsenic toxicity. Toxicol. Appl. Pharmacol.

[26] Drobná Z, Walton F.S, Paul D.S, Xing W, Thomas D.J, Stýblo M (2010). Metabolism of arsenic in human liver:The role of membrane transporters. Arch. Toxicol.

[27] Yan Y, Ye J, Zhang X, Xue X.M, Zhu Y.G (2019). Structural insight into the catalytic mechanism of arsenate reductase from *Synechocystis* sp. PCC 6803. In:Environmental Arsenic in a Changing World.

[28] Maimaitiyiming Y, Wang C, Xu S, Islam K, Chen Y.J, Yang C, Wang Q.Q, Naranmandura H (2018). Role of arsenic (+3 oxidation state) methyltransferase in arsenic mediated APL treatment:An *in vitro* investigation. Metallomics.

[29] Ding L, Saunders R.J, Drobná Z, Walton F.S, Xun P, Thomas D.J, Stýblo M (2012). Methylation of arsenic by recombinant human wild-type arsenic (+3 oxidation state) methyltransferase and its methionine 287 threonine (M287T) polymorph:Role of glutathione. Toxicol. Appl. Pharmacol.

[30] Ganie S.Y, Javaid D, Hajam Y.A, Reshi M.S (2023). Arsenic toxicity:Sources, pathophysiology and mechanism. Toxicol. Res. (Camb).

[31] Pan H, Zhou L, Zou J, Sun J, You Y, Zhong G, Liao J, Zhang H, Tang Z, Hu L (2024). Arsenic trioxide induces innate immune response and inflammatory response in chicken liver via cGAS-STING/NF-kB pathway. Comp. Biochem. Physiol. C Toxicol. Pharmacol.

[32] Sies H (2020). Oxidative stress:Concept and some practical aspects. Antioxidants (Basel).

[33] Manna S.K, Roy S.K, Naskar J.P, Mukherjee A.K (2017). Status of urinary malonaldehyde as a biomarker of oxidative stress among population exposed to arsenic contaminated drinking water in endemic area of West Bengal. Indian J. Environ. Protect.

[34] Kaushal G.P, Chandrashekar K, Juncos L.A (2019). Molecular interactions between reactive oxygen species and autophagy in kidney disease. Int. J. Mol. Sci.

[35] Halliwell B (2024). Understanding mechanisms of antioxidant action in health and disease. Nat. Rev. Mol. Cell Biol.

[36] Wang Y, Song M, Wang Q, Guo C, Zhang J, Zhang X, Cui Y, Cao Z, Li Y (2022). PINK1/Parkin-mediated mitophagy is activated to protect against AFB1-induced kidney damage in mice. Chem. Biol. Interact.

[37] Lin Q, Li S, Jiang N, Shao X, Zhang M, Jin H, Zhang Z, Shen J, Zhou Y, Zhou W, Gu L, Lu R, Ni Z (2019). PINK1-parkin pathway of mitophagy protects against contrast-induced acute kidney injury via decreasing mitochondrial ROS and NLRP3 inflammasome activation. Redox Biol.

[38] Wei M, Liu J, Xu M, Rui D, Xu S, Feng G, Ding Y, Li S, Guo S (2016). Divergent effects of arsenic on NF-kB signaling in different cells or tissues:A systematic review and meta-analysis. Int. J. Environ. Res. Public Health.

[39] Hu Y, Li J, Lou B, Wu R, Wang G, Lu C, Wang H, Pi J, Xu Y (2020). The role of reactive oxygen species in arsenic toxicity. Biomolecules.

[40] Xing P, Zhang Y, Chi Q, Li S (2022). Zinc alleviates arsenic-induced inflammation and apoptosis in the head kidney of common carp by inhibiting oxidative stress and endoplasmic reticulum stress. Biol. Trace Elem. Res.

[41] Hajam Y.A, Rani R, Ganie S.Y, Sheikh T.A, Javaid D, Qadri S.S, Pramodh S, Alsulimani A, Alkhanani M.F, Harakeh S, Hussain A, Haque S, Reshi M.S (2022). Oxidative stress in human pathology and aging:Molecular mechanisms and perspectives. Cells.

[42] Chiu C.Y, Chung M.N, Lan K.C, Yang R.S, Liu S.H (2020). Exposure of low-concentration arsenic induces myotube atrophy by inhibiting an Akt signaling pathway. Toxicol. In Vitro.

[43] Fu S.C, Lin J.W, Liu J.M, Liu S.H, Fang K.M, Su C.C, Hsu R.J, Wu C.C, Huang C.F, Lee K.I, Chen Y.W (2021). Arsenic induces autophagy-dependent apoptosis via Akt inactivation and AMPK activation signaling pathways leading to neuronal cell death. Neurotoxicology.

[44] Wadgaonkar P, Chen F (2021). Connections between endoplasmic reticulum stress-associated unfolded protein response mitochondria and autophagy in arsenic-induced carcinogenesis. Semin. Cancer Biol.

[45] Wadgaonkar P, Bi Z, Wan J, Fu Y, Zhang Q, Almutairy B, Zhang W, Qiu Y, Thakur C, Hüttemann M, Chen F (2022). Arsenic activates the ER stress-associated unfolded protein response via the activating transcription factor 6 in human bronchial epithelial cells. Biomedicines.

[46] Yan M, Shu S, Guo C, Tang C, Dong Z (2018). Endoplasmic reticulum stress in ischemic and nephrotoxic acute kidney injury. Ann. Med.

[47] Mashayekhi-Sardoo H, Rezaee R, Yarmohammadi F, Karimi G (2024). Targeting endoplasmic reticulum stress by natural and chemical compounds ameliorates cisplatin-induced nephrotoxicity:A review. Biol. Trace Elem. Res.

[48] Tryndyak V.P, Borowa-Mazgaj B, Steward C.R, Beland F.A, Pogribny I.P (2020). Epigenetic effects of low-level sodium arsenite exposure on human liver HepaRG cells. Arch. Toxicol.

[49] Shen F, Zhuang S (2022). Histone acetylation and modifiers in renal fibrosis. Front. Pharmacol.

[50] Wang Q, Fan X, Sheng Q, Yang M, Zhou P, Lu S, Gao Y, Kong Z, Shen N, Lv Z, Wang R (2023). N6-methyladenosine methylation in kidney injury. Clin Epigenetics.

[51] Wang Z, Uddin M.B, Wang P.S, Liu Z, Barzideh D, Yang C (2023). Up-regulation of RNA m6A methyltransferase like-3 expression contributes to arsenic and benzo [a] pyrene co-exposure-induced cancer stem cell-like property and tumorigenesis. Toxicol. Appl. Pharmacol.

[52] Imam M.U, Zhang S, Ma J, Wang H, Wang F (2017). Antioxidants mediate both iron homeostasis and oxidative stress. Nutrients.

[53] Li T, Mao C, Wang X, Shi Y, Tao Y (2020). Epigenetic crosstalk between hypoxia and tumor driven by HIF regulation. J. Exp. Clin. Cancer Res.

[54] Jiang X, Stockwell B.R, Conrad M (2021). Ferroptosis:Mechanisms, biology and role in disease. Nat. Rev. Mol. Cell Biol.

[55] Fujii J, Imai H (2024). Oxidative metabolism as a cause of lipid peroxidation in the execution of ferroptosis. Int. J. Mol. Sci.

[56] Zhang S, Cao S, Zhou H, Li L, Hu Q, Mao X, Ji S (2022). Realgar-induced nephrotoxicity via ferroptosis in mice. Appl. Toxicol.

[57] Wang Y, Bi R, Quan F, Cao Q, Lin Y, Yue C, Cui X, Yang H, Gao X, Zhang D (2020). Ferroptosis involves in renal tubular cell death in diabetic nephropathy. Eur. J. Pharmacol.

[58] Roy A, Manna P, Sil P.C (2009). Prophylactic role of taurine on arsenic mediated oxidative renal dysfunction via MAPKs/NF-k B and mitochondria dependent pathways. Free Radic. Res.

[59] Zhang X, Li X (2022). Abnormal iron and lipid metabolism mediated ferroptosis in kidney diseases and its therapeutic potential. Metabolites.

[60] Li W, Liang L, Liu S, Yi H, Zhou Y (2023). FSP1:A key regulator of ferroptosis. Trends Mol. Med.

[61] Song Z, Hei T.K, Gong X (2025). Tetramethylpyrazine attenuates sodium arsenite-induced acute kidney injury by improving the autophagic flux blockade via a YAP1-Nrf2-p62-dependent mechanism. Int. J. Biol. Sci.

[62] Xu G, Peng H, Yao R, Yang Y, Li B (2024). TFEB and TFE3 cooperate in regulating inorganic arsenic-induced autophagy-lysosome impairment and immuno-dysfunction in primary dendritic cells. Cell Biol. Toxicol.

[63] Valentijn F.A, Falke L.L, Nguyen T.Q, Goldschmeding R (2018). Cellular senescence in the aging and diseased kidney. J. Cell Commun. Signal.

[64] Chang Y.W, Singh K.P (2019). Arsenic induces fibrogenic changes in human kidney epithelial cells potentially through epigenetic alterations in DNA methylation. J. Cell. Physiol.

[65] Gu Y, Qiu Y, Li Y, Wen W (2024). Research progress on the regulatory mechanism of cell senescence in arsenic toxicity:A systematic review. Toxicol. Res.

[66] Xiong Y.B, Huang W.Y, Ling X, Zhou S, Wang X.X, Li X.L, Zhou L.L (2024). Mitochondrial calcium uniporter promotes kidney aging in mice through inducing mitochondrial calcium-mediated renal tubular cell senescence. Acta Pharmacol. Sin.

[67] Dong L, Luo P, Zhang A (2024). Intestinal microbiota dysbiosis contributes to the liver damage in subchronic arsenic-exposed mice:Intestinal microbiota imbalance of arsenic-induced hepatotoxicity in mice. Acta Biochim. Biophys. Sin.

[68] Orozco H, Devesa V, Domene A, Vélez D, Monedero V, Zúñiga M, Rodríguez-Viso P (2023). Impact of chronic exposure to arsenate through drinking water on the intestinal barrier. Chem. Res. Toxicol.

[69] Huang L, Ye Z, Zhao Q, Li Y, Yu Z.G, Zhang W (2023). Role of microbial microbes in arsenic bioaccumulation and biotransformation in mice. Toxicol. Appl. Pharmacol.

[70] Wu S, Zhong G, Su Q, Hu T, Rao G, Li T, Wu Y, Ruan Z, Zhang H, Tang Z, Hu L (2024). Arsenic induced neurotoxicity in the brain of ducks:The potential involvement of the gut-brain axis. Trace Elem. Med. Biol.

[71] Banerjee A, Chatterji U (2024). Prevalence of perturbed gut microbiota in pathophysiology of arsenic-induced anxiety-and depression-like behaviour in mice. Chemosphere.

[72] Wu H, Wu R, Chen X, Geng H, Hu Y, Gao L, Fu J, Pi J, Xu Y (2022). Developmental arsenic exposure induces dysbiosis of gut microbiota and disruption of plasma metabolites in mice. Toxicol. Appl. Pharmacol.

[73] Liu Q, Liu Y, Zhang J, Guan Y, Zhou Q, Yan Y, Li W, An J, He M (2024). Gut microbiota deficiency aggravates arsenic-induced toxicity by affecting bioaccumulation and biotransformation in C57BL/6J mice. Food Chem. Toxicol.

[74] Yang Y, Chi L, Liu C.W, Hsiao Y.C, Lu K (2023). Chronic arsenic exposure perturbs gut microbiota and bile acid homeostasis in mice. Chem. Res. Toxicol.

[75] Agusa T, Fujihara J, Takeshita H, Iwata H (2011). Individual variations in inorganic arsenic metabolism associated with AS3MT genetic polymorphisms. Int. J. Mol. Sci.

[76] Schläwicke Engström K, Nermell B, Concha G, Strömberg U, Vahter M, Broberg K (2009). Arsenic metabolism is influenced by polymorphisms in genes involved in one-carbon metabolism and reduction reactions. Mutat. Res.

[77] Steinmaus C, Moore L.E, Shipp M, Kalman D, Rey O.A, Biggs M.L, Hopenhayn C, Bates M.N, Zheng S, Wiencke J.K, Smith A.H (2007). Genetic polymorphisms in MTHFR 677 and 1298, GSTM1 and T1, and metabolism of arsenic. J. Toxicol. Environ. Health. Part A.

[78] Kobayashi Y, Agusa T (2019). Arsenic metabolism and toxicity in humans and animals:racial and species differences. In:Arsenic Contamination in Asia:Biological Effects and Preventive Measures.

[79] Schläwicke Engström K, Broberg K, Concha G, Nermell B, Warholm M, Vahter M (2007). Genetic polymorphisms influencing arsenic metabolism:evidence from Argentina. Environ. Health Perspect.

[80] Pierce B.L, Kibriya M.G, Tong L, Jasmine F, Argos M, Roy S, Paul-Brutus R, Rahaman R, Rakibuz-Zaman M, Parvez F, Ahmed A (2012). Genome-wide association study identifies chromosome 10q24. 32 variants associated with arsenic metabolism and toxicity phenotypes in Bangladesh. PLoS Genet.

[81] Engström K, Vahter M, Mlakar S.J, Concha G, Nermell B, Raqib R, Cardozo A, Broberg K (2011). Polymorphisms in arsenic (+III oxidation state) methyltransferase (AS3MT) predict gene expression of AS3MT as well as arsenic metabolism. Environ. Health Perspect.

[82] Lesseur C, Gilbert-Diamond D, Andrew A.S, Ekstrom R.M, Li Z, Kelsey K.T, Marsit C.J, Karagas M.R (2012). A case-control study of polymorphisms in xenobiotic and arsenic metabolism genes and arsenic-related bladder cancer in New Hampshire. Toxicol. Lett.

[83] Luo L, Li Y, Gao Y, Zhao L, Feng H, Wei W, Qiu C, He Q, Zhang Y, Fu S, Sun D (2018). Association between arsenic metabolism gene polymorphisms and arsenic-induced skin lesions in individuals exposed to high-dose inorganic arsenic in northwest China. Sci. Rep.

[84] Roy J, Mukhopadhyay A, Chanda S, Mazumder D, Chakraborty T (2021). Modification of DNMTs gene expressions by GST O1 and GST O2 polymorphism in chronic arsenic exposed people with and without malignancy from West Bengal, India. Expos. Health.

[85] Liao P.J, Hsu K.H, Chiou H.Y, Chen C.J, Lee C.H (2021). Joint effects of genomic markers and urinary methylation capacity associated with inorganic arsenic metabolism on the occurrence of cancers among residents in arseniasis-endemic areas:A cohort subset with average fifteen-year follow-up. Biomed. J.

[86] Ren M, Li J, Xu Z, Nan B, Gao H, Wang H, Lin Y, Shen H (2024). Arsenic exposure induced renal fibrosis via regulation of mitochondrial dynamics and the NLRP3-TGF-b1/SMAD signaling pathway. Environ. Toxicol.

[87] Huang Z, Guo L, Chen X, Sun J, Ye Y, Sheng L, Zhang Y, Zhou J, Ji J, Sun X (2023). Long-term chronic food-derived arsenic exposure induce the urinary system metabolic dysfunction in mice. Sci. Total Environ.

[88] Fatima G, Raza A.M, Dhole P (2025). Heavy metal exposure and its health implications:A comprehensive review. Indian J. Clin. Biochem.

[89] Genchi G, Lauria G, Catalano A, Carocci A, Sinicropi M.S (2022). Arsenic:A review on a great health issue worldwide. Appl. Sci.

[90] Bhadauria S, Flora S.J (2007). Response of arsenic-induced oxidative stress, DNA damage, and metal imbalance to combined administration of DMSA and monoisoamyl-DMSA during chronic arsenic poisoning in rats. Cell Biol. Toxicol.

[91] Bhattacharya S (2023). Herbal options for arsenic toxicity mitigation:An appraisal. In:Arsenic Toxicity Remediation:Biotechnological Approaches. Springer Nature Switzerland. Cham.

[92] Norouzzadeh M, Kalantar H, Khorsandi L, Mohtadi S, Khodayar M.J (2024). Betaine ameliorates arsenic-induced kidney injury in mice by mitigating oxidative stress-mediated inflammation. Arch. Biochem. Biophys.

[93] Akaras N, Gur C, Kucukler S, Kandemir F.M (2023). Zingerone reduces sodium arsenite-induced nephrotoxicity by regulating oxidative stress, inflammation, apoptosis and histopathological changes. Chem. Biol. Interact.

[94] Mehrzadi S, Goudarzi M, Fatemi I, Basir Z, Malayeri A, Khalili H (2021). Chrysin attenuates sodium arsenite-induced nephrotoxicity in rats by suppressing oxidative stress and inflammation. Tissue Cell.

[95] Wan F, Zhong G, Wu S, Jiang X, Liao J, Zhang X, Zhang H, Mehmood K, Tang Z, Hu L (2021). Arsenic and antimony co-induced nephrotoxicity via autophagy and pyroptosis through ROS-mediated pathway *in vivo* and *in vitro*
*Ecotoxicol. Environ*. Saf.

[96] Sosa C, Guillén N, Lucea S, Sorribas V (2020). Effects of oral exposure to arsenite on arsenic metabolism and transport in rat kidney. Toxicol. Lett.

[97] Jin W, Xue Y, Xue Y, Han X, Song Q, Zhang J, Li Z, Cheng J, Guan S, Sun S, Chu L (2020). Tannic acid ameliorates arsenic trioxide-induced nephrotoxicity, contribution of NF-kB and Nrf2 pathways. Biomed. Pharmacother.

[98] Vadizadeh A, Salehcheh M, Kalantar H, Khorsandi L, Rashno M, Mahdavinia M (2024). Cannabidiol attenuates arsenic-induced nephrotoxicity via the NOX4 and NF-kB pathways in mice. Res. Pharm. Sci.

[99] Sharma S, Kaur T, Sharma A.K, Singh B, Pathak D, Yadav H.N, Singh A.P (2022). Betaine attenuates sodium arsenite-induced renal dysfunction in rats. Drug Chem. Toxicol.

[100] Liu S, Liu Y, Li J, Wang M, Chen X, Gan F, Wen L, Huang K, Liu D (2023). Arsenic exposure-induced acute kidney injury by regulating SIRT1/PINK1/mitophagy axis in mice and in HK-2 cells. J. Agric. Food Chem.

[101] Khodayar M.J, Shirani M, Shariati S, Khorsandi L, Mohtadi S (2024). Antioxidant and anti-inflammatory potential of vanillic acid improves nephrotoxicity induced by sodium arsenite in mice. Int. J. Environ. Health Res.

[102] Abdel-Wahab B.A, El-Shoura E.A, Habeeb M.S, Zafaar D (2023). Febuxostat alleviates arsenic trioxide-induced renal injury in rats:Insights on the crosstalk between NLRP3/TLR4. Sirt-1/NF-kB/TGF-b signaling pathways and miR-23b-3p miR-181a-5b expression. Biochem. Pharmacol.

[103] Liu P, Xue Y, Zheng B, Liang Y, Zhang J, Shi J, Chu X, Han X, Chu L (2020). Crocetin attenuates the oxidative stress, inflammation and apoptosis in arsenic trioxide-induced nephrotoxic rats:Implication of PI3K/AKT pathway. Int. Immunopharmacol.

[104] Sharma A.K, Kaur J, Kaur T, Singh B, Yadav H.N, Pathak D, Singh A.P (2021). Ameliorative role of bosentan, an endothelin receptor antagonist, against sodium arsenite-induced renal dysfunction in rats. Environ. Sci. Pollut. Res.

[105] Das K, Rao L.V.M (2022). The role of microRNAs in inflammation. Int. J. Mol. Sci.

[106] Di W, Li Y, Zhang L, Zhou Q, Fu Z, Xi S (2024). The Hippo-YAP1/HIF-1a pathway mediates arsenic-induced renal fibrosis. Environ. Res. J.

[107] Sung J.M, Chang W.H, Liu K.H, Chen C.Y, Mahmudiono T, Wang W.R, Hsu H.C, Li Z.Y, Chen H.L (2022). The effect of co-exposure to glyphosate, cadmium, and arsenic on chronic kidney disease. Expos. Health.

[108] Tian L, Shao X, Xie Y, Wang Q, Che X, Zhang M, Xu W, Xu Y, Mou S, Ni Z (2017). Kidney injury molecule-1 is elevated in nephropathy and mediates macrophage activation via the Mapk signalling pathway. Cell. Physiol. Biochem.

[109] Hasan H.R, Yousif Al-Fatlawi A.C, Al-Obaidy Q.M (2024). Kidney injury molecule-1 (KIM-1), neutrophil gelatinase associated lipocalin (NGAL), and CRP:A potential biomarker for early prediction of Acute Kidney Injury (AKI) in pediatric male patients. Fam. Med. Prim. Care Rev.

[110] Yin W, Kumar T, Lai Z, Zeng X, Kanaan H.D, Li W, Zhang P.L (2019). Kidney injury molecule-1, a sensitive and specific marker for identifying acute proximal tubular injury, can be used to predict renal functional recovery in native renal biopsies. Int. Urol. Nephrol.

[111] Ismail O.Z, Zhang X, Wei J, Haig A, Denker B.M, Suri R.S, Sener A, Gunaratnam L (2015). Kidney injury molecule-1 protects against Ga12 activation and tissue damage in renal ischemia-reperfusion injury. Am. J. Pathol.

[112] Cruz D.N, Soni S, Ronco C (2009). NGAL and cardiac surgery-associated acute kidney injury. Am. J. Kidney Dis.

[113] Bellos I, Fitrou G, Daskalakis G, Perrea D.N, Pergialiotis V (2018). Neutrophil gelatinase-associated lipocalin as predictor of acute kidney injury in neonates with perinatal asphyxia:A systematic review and meta-analysis. Eur. J. Pediatr.

[114] Chai L, Zeng J, Gong L, Li Z, Wang F, Liu Z, Fan W, Shen B (2024). The relationship between serum levels of LOX-1, hs-cTnT, NGAL, and renal function, and their diagnostic value in patients with chronic kidney disease:A retrospective study. BMC Nephrol.

[115] Novak R, Salai G, Hrkac S, Vojtusek I.K, Grgurevic L (2023). Revisiting the role of NAG across the continuum of kidney disease. Bioengineering (Basel).

[116] Cheng P, Miao Q, Huang J, Li J, Pu K (2020). Multiplex optical urinalysis for early detection of drug-induced kidney injury. Anal. Chem.

[117] Qiu X, Miao Y, Geng X, Zhou X, Li B (2020). Evaluation of biomarkers for *in vitro* prediction of drug-induced nephrotoxicity in RPTEC/TERT1 cells. Toxicol. Res.

[118] Chin W.S, Hung W.L, Say Y.H, Chien L.C, Chen Y.C, Lo Y.P, Liao K.W (2024). The influence of exposure to inorganic arsenic and other arsenic species on early renal impairment among young adults in Taiwan. Environ. Pollut.

[119] Choi S.H, Choi K.H, Won J.U, Kim H (2023). Impact of multi-heavy metal exposure on renal damage indicators in Korea:An analysis using Bayesian Kernel Machine Regression. Medicine (Baltimore).

[120] Quan J, Li Y, Shen M, Lu Y, Yuan H, Yi B, Chen X, Huang Z (2023). Coexposure to multiple metals and renal tubular damage:A population-based cross-sectional study in China's rural regions. Environ. Sci. Pollut. Res. Int.

[121] Amatruda J.G, Estrella M.M, Garg A.X, Thiessen-Philbrook H, McArthur E, Coca S.G, Parikh C.R, Shlipak M.G, TRIBE-AKI Consortium (2021). Urine alpha-1-microglobulin levels and acute kidney injury, mortality, and cardiovascular events following cardiac surgery. Am. J. Nephrol.

[122] Liu Q, Lei Z (2023). The role of microRNAs in arsenic-induced human diseases:A review. J. Agric. Food Chem.

[123] Shihana F, Wong W.K.M, Joglekar M.V, Mohamed F, Gawarammana I.B, Isbister G.K, Hardikar A.A, Seth D, Buckley N.A (2021). Urinary microRNAs as non-invasive biomarkers for toxic acute kidney injury in humans. Sci. Rep.

[124] Ashrafizadeh M, Ahmadi Z, Samarghandian S, Mohammadinejad R, Yaribeygi H, Sathyapalan T, Sahebkar A (2020). MicroRNA-mediated regulation of Nrf2 signaling pathway:Implications in disease therapy and protection against oxidative stress. Life Sci.

[125] Chen X, Yan X, Tang X, Wang Y, Zhang X, Cao X, Ran X, Ma G, Hu T, Qureshi A, Luo P, Shen L (2024). Study on the mechanism of arsenic-induced renal injury based on SWATH proteomics technology. J. Trace Elem. Med. Biol.

[126] Zhang S, Li C, Feng T, Cao S, Zhou H, Li L, Hu Q, Mao X, Ji S (2021). A metabolic profiling study of realgar-induced acute kidney injury in mice. Front. Pharmacol.

[127] Ghasemiyeh P, Vazin A, Zand F, Haem E, Karimzadeh I, Azadi A, Masjedi M, Sabetian G, Nikandish R, Mohammadi-Samani S (2022). Pharmacokinetic assessment of vancomycin in critically ill patients and nephrotoxicity prediction using individualized pharmacokinetic parameters. Front. Pharmacol.

[128] Gong Y, Teng D, Wang Y, Gu Y, Wu Z, Li W, Tang Y, Liu G (2022). In silico prediction of potential drug-induced nephrotoxicity with machine learning methods. J. Appl. Toxicol.

[129] Wang Q, Lu J, Fan K, Xu Y, Xiong Y, Sun Z, Zhai M, Zhang Z, Zhang S, Song Y, Luo J, You M, Guo M, Zhang X (2022). High-throughput “read-on-ski”automated imaging and label-free detection system for toxicity screening of compounds using personalised human kidney organoids. J. Zhejiang Univ. Sci. B.

[130] Connolly P, Stapleton S, Mosoyan G, Fligelman I, Tonar Y.C, Fleming F, Donovan M.J (2021). Analytical validation of a multi-biomarker algorithmic test for prediction of progressive kidney function decline in patients with early-stage kidney disease. Clin. Proteomics.

[131] Owens E, Tan K.S, Ellis R, Del Vecchio S, Humphries T, Lennan E, Vesey D, Healy H, Hoy W, Gobe G (2020). Development of a biomarker panel to distinguish risk of progressive chronic kidney disease. Biomedicines.

[132] Valente M.J, Rocha S, Lousa I, Reis F, Nunes S, Viana S, Preguiça I, Mira F, Nogueira R, Coimbra S, Catarino C (2020). P0739 Panel of sensitive biomarkers of the primary response to renal injury for an early diagnosis of chronic kidney disease. Nephrol. Dial. Transplant.

[133] Lousa I, Valente M. J, Rocha S, D. Viana S, Preguica I, Mira F, Nogueira R, Coimbra S, Catarino C, Rocha-Pereira P, Bronze-da-Rocha E (2021). MO472 A preliminary study of potential biomarkers for early diagnosis in chronic kidney disease. Nephrol Dial. Transplant.

[134] Kohl K, Herzog E, Dickneite G, Pestel S (2020). Evaluation of urinary biomarkers for early detection of acute kidney injury in a rat nephropathy model. J. Pharmacol. Toxicol. Methods.

[135] Qin Q, Lu H, Zhu Z, Sui M, Qiu Y, Yin D (2021). Reduction in arsenic exposure by domestic water purification devices in Shanghai area and related health risk assessment. Water.

[136] Ahsan H, Pierce B (2019). Genetic susceptibility and alterations in relation to arsenic exposure metabolism and toxicity. In:Environmental Arsenic in a Changing World.

[137] Chernoff M.B, Delgado D, Tong L, Chen L, Oliva M, Tamayo L.I, Best L.G, Cole S, Jasmine F, Kibriya M.G, Nelson H, Huang L, Haack K, Kent J, Umans J.G, Graziano J, Navas-Acien A, Karagas M.R, Ahsan H, Pierce B.L (2023). Sequencing-based fine-mapping and *in silico* functional characterization of the 10q24.32 arsenic metabolism efficiency locus across multiple arsenic-exposed populations. PLoS Genet.

[138] Mao K, Zhang H, Wang Z, Cao H, Zhang K, Li X, Yang Z (2020). Nanomaterial-based aptamer sensors for arsenic detection. Biosens. Bioelectron.

[139] Li P, Wang Y, Yuan X, Liu X, Liu C, Fu X, Sun D, Dang Y, Holmes D.E (2021). Development of a whole-cell biosensor based on an ArsR-P ars regulatory circuit from Geobacter sulfurreducens. Environ. Sci. Ecotech.

[140] Popescu S.M, Mansoor S, Wani O.A, Kumar S.S, Sharma V, Sharma A, Arya V.M, Kirkham M.B, Hou D, Bolan N, Chung Y.S (2024). Artificial intelligence and IoT driven technologies for environmental pollution monitoring and management. Front. Environ. Sci.

[141] Ahangarpour A, Alboghobeish S, Oroojan A.A, Zeidooni L, Samimi A, Afshari G (2018). Effects of combined exposure to chronic high-fat diet and arsenic on thyroid function and lipid profile in male mouse. Biol. Trace Elem. Res.

[142] Yu H, Liu S, Li M, Wu B (2016). Influence of diet, vitamin, tea, trace elements and exogenous antioxidants on arsenic metabolism and toxicity. Environ. Geochem. Health.

[143] Barreto F.C, Barreto D.V, Canziani M.E.F (2017). Uremia retention molecules and clinical outcomes. Contrib. Nephrol.

[144] Ghosh J, Sil P.C (2023). Mechanism for arsenic-induced toxic effects. In:Handbook of Arsenic Toxicology.

[145] Alhawatmeh H, Alshammari S, Rababah J.A (2022). Effects of mindfulness meditation on trait mindfulness, perceived stress, emotion regulation, and quality of life in hemodialysis patients:A randomized controlled trial. Int. J. Nurs. Sci.

[146] Juszczak A.B, Kupczak M, Konecki T (2023). Does vitamin supplementation play a role in chronic kidney disease?. Nutrients.

[147] Baltusnikiene A, Staneviciene I, Jansen E (2023). Beneficial and adverse effects of vitamin E on the kidney. Front. Physiol.

[148] Cepaityte D, Leivaditis K, Varouktsi G, Roumeliotis A, Roumeliotis S, Liakopoulos V (2023). N-Acetylcysteine:More than preventing contrast-induced nephropathy in uremic patients-focus on the antioxidant and anti-inflammatory properties. Int. Urol. Nephrol.

[149] Kamt S.F, Liu J, Yan L.J (2023). Renal-protective roles of lipoic acid in kidney disease. Nutrients.

[150] Shin J.W, Chun K.S, Kim D.H, Kim S.J, Kim S.H, Cho N.C, Na H.K, Surh Y.J (2020). Curcumin induces stabilization of Nrf2 protein through Keap1 cysteine modification. Biochem. Pharmacol.

[151] Ishaq A, Gulzar H, Hassan A, Kamran M, Riaz M, Parveen A, Chattha M.S, Walayat N, Fatima S, Afzal S, Fahad S (2021). Ameliorative mechanisms of turmeric-extracted curcumin on arsenic (As)-induced biochemical alterations, oxidative damage, and impaired organ functions in rats. Environ. Sci. Pollut. Res. Int.

[152] Haleem M.A, Khan S.A, Khan M.K, Ahmad R.S, Naqvi S.A.R, Imran M, Ahmad M.H, Anwar H, Nisa M.U (2021). Potential protective role of curcumin powder to regulate arsenic-induced hepatorenal toxicity and hyperlipidemic metabolic dysfunction in rat model. Pak. J. Pharm. Sci.

[153] Zhu Z, Li J, Song Z, Li T, Li Z, Gong X (2024). Tetramethylpyrazine attenuates renal tubular epithelial cell ferroptosis in contrast-induced nephropathy by inhibiting transferrin receptor and intracellular reactive oxygen species. Clin. Sci. (Lond).

[154] Lin J, Wang Q, Zhou S, Xu S, Yao K (2022). Tetramethylpyrazine:A review on its mechanisms and functions. Biomed. Pharmacother.

[155] Li M, Liu P, Xue Y, Liang Y, Shi J, Han X, Zhang J, Chu X, Chu L (2020). Tannic acid attenuates hepatic oxidative stress, apoptosis and inflammation by activating the Keap1 Nrf2/ARE signaling pathway in arsenic trioxide toxicated rats. Oncol. Rep.

[156] Wang S, Shang S, Lv J, Hou D (2021). Astaxanthin improves renal fibrosis and protecting renal injury in spontaneously hypertensive rats. Latin Am. J. Pharm.

[157] Shen W.C, Chou Y.H, Shi L.S, Chen Z.W, Tu H.J, Lin X.Y, Wang G.J (2021). AST-120 improves cardiac dysfunction in acute kidney injury mice via suppression of apoptosis and proinflammatory NF-kB/ICAM-1 signaling. J. Inflamm. Res.

[158] Wiciński M, Fajkiel-Madajczyk A, Kurant Z, Liss S, Szyperski P, Szambelan M, Gromadzki B, Rupniak I, Słupski M, Sadowska-Krawczenko I (2024). Ashwagandha's multifaceted effects on human health:Impact on vascular endothelium, inflammation, lipid metabolism, and cardiovascular outcomes-a review. Nutrients.

[159] Grunz-Borgmann E, Mossine V, Fritsche K, Parrish A.R (2015). Ashwagandha attenuates TNF-a- and LPS-induced NF-kB activation and CCL2 and CCL5 gene expression in NRK-52E cells. BMC Complement. Altern. Med.

[160] Robles-Osorio M.L, Sabath-Silva E, Sabath E (2015). Arsenic-mediated nephrotoxicity. Renal Fail.

[161] Gong X, Ivanov V.N, Hei T.K (2016). 2,3,5,6-Tetramethylpyrazine (TMP) down-regulated arsenic-induced heme oxygenase-1 and ARS2 expression by inhibiting Nrf2, NF-kB, AP-1 and MAPK pathways in human proximal tubular cells. Arch. Toxicol.

[162] Puri B, Kulkarni Y.A, Gaikwad A.B (2025). Advances in CRISPR-Cas systems for kidney diseases. Prog. Mol. Biol. Transl. Sci.

[163] Tavakolidakhrabadi N, Aulicino F, May C.J, Saleem M.A, Berger I, Welsh G.I (2024). Genome editing and kidney health. Clin. Kidney J.

[164] Liu Z, Gao H, Li G, Yu Y, Cui M, Peng H, Guan X, Zhang X, Zhang Z, Shen X, Chen S, Li D, Chen L, Xiao Y, Chen W, Liu L, Wang Q (2025). Genome-wide CRISPR-based screen identifies E2F transcription factor 1 as a regulator and therapeutic target of aristolochic acid-induced nephrotoxicity. Environ. Int.

[165] Cruz N.M, Freedman B.S (2018). CRISPR gene editing in the kidney. Am. J. Kidney Dis.

[166] Li S, Dong X, Xu L, Wu Z (2023). nephroprotective effects of selenium nanoparticles against sodium arsenite-induced damages. Int. J. Nanomedicine.

[167] Yang Y, Chi L, Lai Y, Hsiao Y.C, Ru H, Lu K (2021). The gut microbiome and arsenic-induced disease-iAs metabolism in mice. Curr. Environ. Health Rep.

[168] Yang D, Xia X, Xi S (2024). Salvianolic acid A attenuates arsenic-induced ferroptosis and kidney injury via HIF-2a/DUOX1/GPX4 and iron homeostasis. Sci. Total Environ.

[169] Khalid S, Shahid M, Bibi I, Natasha N, Murtaza B, Tariq T.Z, Naz R, Shahzad M, Hussain M.M, Niazi N.K (2022). Global arsenic contamination of groundwater, soil and food crops and health impacts. In:Global Arsenic Hazard:Ecotoxicology and Remediation.

[170] Kapwata T, Wright C.Y, Reddy T, Street R, Kunene Z, Mathee A (2023). Relations between personal exposure to elevated concentrations of arsenic in water and soil and blood arsenic levels amongst people living in rural areas in Limpopo, South Africa. Environ. Sci. Pollut. Res.

[171] Mar Wai K, Umezaki M, Mar O, Umemura M, Watanabe C (2019). Arsenic exposure through drinking Water and oxidative stress Status:A cross-sectional study in the Ayeyarwady region, Myanmar. J. Trace Elem. Med. Biol.

[172] Khan K.M, Chakraborty R, Bundschuh J, Bhattacharya P, Parvez F (2020). Health effects of arsenic exposure in Latin America:An overview of the past eight years of research. Sci. Total Environ.

[173] Benchrih R, Buekers J, Demoury C, Martins C, Purece A, Thomsen S, Waegeneers N, Clercq E, Devleesschauwer B (2024). Estimation of environmental burden of disease related to arsenic exposure in European populations. Eur. J. Public Health.

[174] Oberoi S, Devleesschauwer B, Gibb H, Barchowsky A (2019). Global burden of cancer and coronary heart disease resulting from dietary exposure to arsenic, 2015. Environ. Res. J.

[175] Chen Q, Costa M (2021). Arsenic:A global environmental challenge. Annu. Rev. Pharmacol. Toxicol.

[176] Kumar A, Kumar K, Ali M, Raj V, Srivastava A, Kumar M, Niraj P.K, Kumar M, Kumar R, Kumar D, Bishwapriya A (2024). Severe disease burden and the mitigation strategy in the arsenic-exposed population of Kaliprasad Village in Bhagalpur District of Bihar, India. Biol. Trace Elem. Res.

[177] Thakur M, Rachamalla M, Niyogi S, Datusalia A.K, Flora S.J.S (2021). Molecular mechanism of arsenic-induced neurotoxicity including neuronal dysfunctions. Int. J. Mol. Sci.

[178] Xu Y, Zou Z, Liu Y, Wang Q, Sun B, Zeng Q, Liu Q, Zhang A (2020). miR-191 is involved in renal dysfunction in arsenic-exposed populations by regulating inflammatory response caused by arsenic from burning arsenic-contaminated coal. Hum. Exp. Toxicol.

[179] Li Y, Zhong G, He T, Quan J, Liu S, Liu Z, Tang Z, Yu W (2023). Effect of arsenic and copper in kidney of mice:Crosstalk between Nrf2/Keap1 pathway in apoptosis and pyroptosis. Ecotoxicol. Environ. Saf.

[180] Nail A.N, Xu M, Bastick J.C, Patel D.P, Rogers M.N, States J.C (2023). Arsenic and human health:New molecular mechanisms for arsenic-induced cancers. Curr. Pollut. Rep.

